# Association Between Basal Metabolic Rate and All-Cause Mortality in a Prospective Cohort of Southern Chinese Adults

**DOI:** 10.3389/fphys.2021.790347

**Published:** 2022-01-04

**Authors:** Fengyu Han, Feng Hu, Tao Wang, Wei Zhou, Linjuan Zhu, Xiao Huang, Huihui Bao, Xiaoshu Cheng

**Affiliations:** ^1^The Department of Cardiovascular Medicine, The Second Affiliated Hospital of Nanchang University, Nanchang, China; ^2^Jiangxi Provincial Cardiovascular Disease Clinical Medical Research Center, Nanchang, China; ^3^Center for Prevention and Treatment of Cardiovascular Diseases, The Second Affiliated Hospital of Nanchang University, Nanchang, China

**Keywords:** basal metabolic rate, all-cause mortality, Chinese, adults, aging

## Abstract

**Objective:** The aim of this study was to assess the relationship between basal metabolic rate (BMR) and all-cause mortality in southern Chinese adults.

**Methods:** We prospectively examined the relationship between BMR and all-cause mortality in 12,608 Southern Chinese adults with age ≥ 35 years who participated in the National Key R&D Program from 2013–2014 to 2019–2020. Cox proportional hazard models were used to examine the association between BMR and all-cause mortality.

**Results:** A total of 809 deaths (including 478 men and 331 women) occurred during a median follow-up period of 5.60 years. All-cause mortality was higher in elderly individuals than in non-elderly individuals (11.48 vs. 2.04%, *P* < 0.001) and was higher in male subjects than in female subjects (9.84 vs. 4.56%, *P* < 0.001). There was a significantly inverse relationship between BMR levels and all-cause mortality in elderly male individuals (adjusted-HR per SD increase: 0.80, 95% CI: 0.70–0.91, *P* < 0.001). Compared with BMR levels ≤ 1,115 kcal/day, there was lower all-cause mortality in third and highest BMR quartiles in the elderly male subjects (adjusted-HR: 0.71, 95% CI: 0.53–0.95, *P* = 0.022; adjusted-HR: 0.60, 95% CI: 0.43–0.84, *P* = 0.003, respectively).

**Conclusion:** An elevated BMR was independently inversely associated with all-cause mortality in elderly male subjects in a southern Chinese population.

## Introduction

The question of why we age and finally die has been a central subject in the life, medical, and health sciences. The idea that energy expenditure may be related to aging and longevity is appealing. At the beginning of the twentieth century, scientists noticed that larger, longer-lived animals had lower basal metabolic rate (BMR), and specifically that the product of their metabolism (per gram) and lifespan was essentially constant (Rubner, 1908). These data formed a cornerstone of the rate-of-living hypothesis (Lints, 1989) and the free radical damage theory (Beckman and Ames, 1998) of aging, both of which put forward that the longevity of different animal species is inversely proportional to their energy expenditure. Production of damaging reactive oxygen species from metabolism is thought to cause greater oxidative stress and decreased longevity (Lints, 1989; Beckman and Ames, 1998; Brys et al., 2007; Monaghan et al., 2009).

However, this theory is more confusing among endotherms. More recent analyses suggested that there is no inter-specific relevance between BMR and longevity if only the confounding effects of body composition, such as body size or body fatness, are taken into consideration (Speakman, 2005; Duarte and Speakman, 2014). Surprisingly, comparisons across major classes revealed a positive association. For instance, birds combined unusual longevity relative to mammals with elevated resting metabolic rates (Holmes et al., 2001; Furness and Speakman, 2008; Boyce et al., 2020) and exceptional resistance to oxidative damage (Ogburn et al., 2001; Barja et al., 2020). Moreover, bats and marsupials, which have higher BMR than the eutherian mammals, also generally live longer (Austad and Fischer, 1991). Within species, individual mice with higher BMR had greater mitochondrial uncoupling and live longer (Speakman et al., 2004).

Despite longstanding controversies from animal studies on the relationship between BMR and longevity (Speakman, 2005), studies investigating that whether BMR is a risk factor for mortality in humans were extremely limited (Ruggiero et al., 2008; Jumpertz et al., 2011; Schrack et al., 2014). In a clinical setting, BMR may be considered as the energy required to maintain structural and functional homeostasis at rest, in fasting and thermoneutral conditions. BMR declines with age and the rate of decline accelerates at older ages (Rizzo et al., 2005). In the Baltimore Longitudinal Study of Aging (BLSA) based on over 3,000 individuals over a 40-year follow-up, BMR was an independent risk factor for increased mortality (Ruggiero et al., 2008; Schrack et al., 2014). Higher metabolic rates as reflected by 24-h energy expenditure or BMR predict early natural mortality in Pima Indians with a mean follow-up time of 11.1 years (Jumpertz et al., 2011).

In consideration of the complicated relationship between the BMR and mortality among different species, regions, and ethnicities, the hypothesis that BMR is a risk factor for mortality in humans needs further confirmation. Therefore, we prospectively examined the relationship between BMR and all-cause mortality in Chinese adults.

## Materials and Methods

### Study Design and Population

Our study was supported by the National Key R&D Program in the Twelfth Five-year Plan (No. 2011BAI11B01) from the Chinese Ministry of Science. This program was aimed to monitor the nutrition and health condition of rural residents in China. Details in regard to the background, aims, methods, and design of the trial have been described in detail in previous publications (Wang et al., 2014; Hu et al., 2017a,b, 2018a,b). In brief, in order to ascertain the current status of hypertension (HT), Jiangxi Province was divided into urban areas and rural areas on the basis of administrative data using a stratified multistage random sampling method. Using the probability proportional to size (PPS) method, four cities in urban areas and four counties in rural areas were selected. Within each urban city and rural county, two districts or townships were selected using the simple random sampling (SRS) method. Three communities or villages were then chosen within each district or township. Finally, a given number of participants from 14 genders and age strata (men/women and ages 15–24, 25–34, 35–44, 45–54, 55–64, 65–74, and ≥ 75 years) were chosen in each community or village. Assuming a design effect of 2.5 and a 17.7% prevalence of HT among the population aged 15 years or older, we estimated that including 15,200 participants would ensure that the average lengths of the 95% CI for the prevalence in the entire population and subpopulations defined by age and gender were less than 0.4 and 1.8%, respectively. Out of 15,364 eligible participants, a total of 15,269 participants completed the investigation between November 2013 and August 2014 (Wang et al., 2014).

From July 1, 2019 to October 1, 2020, we followed 12,608 participants aged ≥ 35 years at baseline. The 12,608 participants were followed up by means of telephone follow-up, death certificate diagnoses from Jiangxi Provincial Center for Disease Control and Prevention (responsible for monitoring provincial causes of death), and a follow-up visit with local public health doctor or village doctor. The median follow-up duration was 5.60 (5.29–5.73) years among all participants. After excluding 181 cases without BMR data, the 1.25% highest and lowest values of BMR (310 cases with extreme value), a final total of 12,117 participants were included in the analysis (Supplementary Figure 1).

### Data Collection Procedure

Participants were required to complete a questionnaire that was developed by the national coordinating center, at Fuwai Cardiovascular Hospital; this questionnaire was conducted through face-to-face interviews by trained staff and included physical measurements using standardized procedures. Data obtained from the questionnaire included personal basic information (e.g., age, gender, area, and education level), behavioral characteristics (e.g., smoking habit, alcohol consumption, physical activity levels, and sleep duration on workdays or non-workdays), medication use, and medical history (e.g., HT, myocardial infarction, and stroke). Current cigarette smokers were defined as having smoked at least one cigarette per day for 6 months or more (Hu et al., 2017b). Current drinkers were defined as drinking alcohol at least one time per week during the previous year (Hu et al., 2017b).

### Anthropometry

The anthropometric examinations included systolic blood pressure (SBP), diastolic blood pressure (DBP), waist circumference, body height, and body weight. BP was measured, with the participant in a sitting position using the Omron HBP-1300 Professional Portable Blood Pressure Monitor (Kyoto, Japan) on the right arm, which was supported at the heart level. After a 5-min rest period, BP was measured three times, and SBP and DBP were calculated as the average of the three measurements. HT was defined as the SBP ≥ 140 mmHg or the DBP ≥ 90 mmHg or on lifestyle or medication treatment for diagnosed HT formerly (Wang et al., 2014).

Waistline was measured using an inelastic measuring tape, with 0.1 cm resolution and a length of 1.5 m. Individuals were at an upright position, with abdomen relaxed at the end of gentle expiration. Body weight without heavy clothing was measured using a weight measurement device (Omron, Kyoto, Japan). Height was measured without shoes using a standard right-angle device and a fixed measurement tape (to the nearest 0.5 cm). BMI was calculated as the weight in kilograms divided by height in meters squared (kg/m^2^). Based on BMI (kg/m^2^), participants were categorized as underweight (<18.5), normal (18.5–23.9), overweight (24–27.9), and obese (≥ 28) (Hu et al., 2017b). In addition, standard protocols and instruments were used. All measurements were taken twice, and the average of the 2 values was adopted. All the investigators were medical students who were systematically trained. The certification requirements for data collection were strict, and a quality assurance program was conducted.

### Basal Metabolic Rate

The BMR was measured using an Omron body fat analysis (Omron Corporation, Tokyo, Japan). All participants rested quietly in a supine position for a minimum of 10 min before BMR measurement. Following a study by Ali et al. (2017), all BMR measurements were performed under standard conditions, i.e., in a quiet environment and at a room temperature of approximately 25°C with the study subjects in a postabsorptive condition after a 12 h fast. All participants were familiarized with the equipment and procedure before BMR measurements to reduce anxiety. BMR was measured by inserting individual age, sex, and anthropometric data into the equipment.

### Mortality Follow-Up

Survival status was ascertained during the follow-up survey between July 1, 2019 and October 1, 2020, assessing whether subjects died and the date of death, completed the study, or were lost to follow-up. Cause of death was ascertained by means of telephone follow-up, death certificate diagnoses from Jiangxi Provincial Center for Disease Control and Prevention, and a follow-up visit with a local public health doctor or village doctor. The causes of death were further categorized into stroke, cardiovascular disease, malignant tumor, respiratory failure, others, and unknown.

### Statistical Analysis

Continuous variables are presented as the mean ± SD and are compared using the one-way ANOVA or the Mann-Whitney *U-*test, depending on whether the quantitative data were consistent with a normal distribution. Categorical variables were expressed as count (percentage), and differences between groups were measured using the chi-square test or Fisher’s exact probability test.

Cox proportional hazard models were used to examine the association between BMR levels and all-cause mortality. The crude model was not adjusted for any confounder. Model I was adjusted for age and sex. Model II was the confounder model. The confounder model screened covariates including age, sex, SBP, DBP, BMI, education level, current smokers and drinkers, physical activity levels, sleep duration on workdays and non-workdays, history of myocardial infarction and stroke, angiotensin-converting enzyme inhibitors (ACEI) or angiotensin receptor blockers (ARB) usage, beta-blocker usage, calcium channel blockers, and diuretic usage. We selected these confounders on the basis of their associations with the outcomes of interest or a change in effect estimates of more than 10% when added to this model. Supplementary Table 1 shows the association of each confounder with all-cause mortality. We considered the confounder model to be the main model. We performed tests for linear trends by entering the median value of each category of the BMR level as a continuous variable in the models. We also performed a sensitivity analysis using a propensity score weighted method excluded or not of subjects with a history of myocardial infarction and stroke or less than 1 year of follow-up. In addition, the generalized additive model and smooth curve fitting (penalized spline method) were used to visually show the relationship between BMR and all-cause mortality grouped by age and sex. We also used propensity-score methods to reduce the effects of confounding. In the inverse-probability-weighted analysis, the predicted probabilities from the propensity-score model were used to calculate the stabilized inverse-probability-weighting weight. Furthermore, the effects of the quartiles of BMR on death events were evaluated with the use of Kaplan-Meier curves (log-rank test) (Xu et al., 2017). In addition, subgroup analysis was executed by stratified and interaction tests to investigate the robustness between BMR levels and all-cause mortality.

All statistical analyses were performed using the statistical package R (The R Foundation)^1^ and the Empower (R; www.empowerstats.com; X&Y Solutions, Inc., Boston, MA, United States). All *P*-values are two-tailed, and *P* < 0.05 was considered statistically significant.

## Results

### Participant Characteristics at Baseline

This analysis included 12,117 Chinese Southern adults (age: 59.02 ± 13.21 years, range 35–97 years; male, 40.08%). The clinical baseline characteristics of the study participants were presented in Table 1 according to the quartiles of BMR. There were significant differences in age, proportion of males and urban residence, SBP, DBP, BMI, waist circumference, education level, prevalence of current smokers and drinkers, physical activity levels, sleep duration on workdays or non-workdays, percentage of current HT, history of myocardial infarction and stroke, and medication usage (antihypertensive medications, ACEIs or ARBs, beta-blockers, calcium channel blockers, and diuretics) among the four BMR categories.

**TABLE 1:** Baseline characteristics of the study population according to quartiles of BMR.

Characteristics	Total subjects	Quartiles of BMR (kcal/day)				*P*-value
		Q1 [787, 1115]	Q2 [1116, 1219]	Q3 [1220, 1367]	Q4 [1368, 1789]	
Number of subjects (n)	12,117	3011	3044	3017	3045	
Age (years)	59.04 ± 13.21	62.53 ± 14.16	58.54 ± 13.05	58.79 ± 12.64	56.35 ± 12.19	<0.001
Male, n (%)	4857 (40.08%)	413 (13.72%)	710 (23.32%)	1224 (40.57%)	2510 (82.43%)	<0.001
SBP (mmHg)	127.95 ± 19.62	127.45 ± 21.02	126.19 ± 19.66	128.11 ± 19.37	130.04 ± 18.17	<0.001
DBP (mmHg)	74.80 ± 10.75	72.47 ± 10.63	73.75 ± 10.35	75.24 ± 10.43	77.70 ± 10.85	<0.001
BMR (kcal/day)	1251.79 ± 190.02	1034.34 ± 66.90	1166.52 ± 31.27	1288.77 ± 43.34	1515.40 ± 107.41	<0.001
BMI (kg/m^2^)	23.07 ± 3.65	21.04 ± 2.91	22.64 ± 2.86	23.61 ± 3.56	24.95 ± 3.97	<0.001
BMI group (kg/m^2^)						<0.001
Underweight (<18.5)	904 (7.47%)	484 (16.02%)	195 (6.41%)	164 (5.44%)	63 (2.07%)	
Normal weight (≥18.5, <24)	6787 (56.05%)	2158 (71.72%)	1974 (64.91%)	1449 (48.06%)	1206 (39.62%)	
Overweight (≥24, <28)	3439 (28.40%)	319 (10.60%)	786 (25.85%)	1104 (36.62%)	1230 (40.41%)	
General obesity (≥28)	979 (8.08%)	50 (1.66%)	86 (2.83%)	298 (9.88%)	545 (17.90%)	
Waist circumference (cm)	79.90 ± 9.14	74.14 ± 7.69	78.15 ± 7.40	81.07 ± 8.11	86.19 ± 8.80	<0.001
Urban residence, n (%)	6199 (51.16%)	1392 (46.23%)	1543 (50.69%)	1611 (53.40%)	1653 (54.29%)	<0.001
Education level, n (%)						<0.001
Primary school or below	7002 (58.51%)	2189 (74.15%)	1861 (61.81%)	1795 (60.40%)	1157 (38.16%)	
Middle school	4577 (38.25%)	727 (24.63%)	1082 (35.93%)	1108 (37.28%)	1660 (54.75%)	
Graduate and above	388 (3.24%)	36 (1.22%)	68 (2.26%)	69 (2.32%)	215 (7.09%)	
Current smokers, n (%)	2338 (19.34%)	219 (7.28%)	358 (11.78%)	623 (20.73%)	1138 (37.50%)	<0.001
Current drinkers, n (%)	3015 (24.96%)	487 (16.23%)	595 (19.60%)	718 (23.89%)	1215 (40.03%)	<0.001
Physical activity levels, n (%)						<0.001
Low	1832 (15.12%)	470 (15.61%)	407 (13.37%)	410 (13.59%)	545 (17.90%)	
Middle	3208 (26.48%)	847 (28.13%)	811 (26.64%)	764 (25.32%)	786 (25.81%)	
High	7047 (58.16%)	1690 (56.13%)	1820 (59.79%)	1829 (60.62%)	1708 (56.09%)	
Sleep duration on workdays (hours)	7.29 ± 1.32	7.24 ± 1.38	7.33 ± 1.29	7.32 ± 1.30	7.26 ± 1.29	0.016
Sleep duration on non-workdays (hours)	7.56 ± 1.36	7.49 ± 1.43	7.61 ± 1.33	7.59 ± 1.35	7.55 ± 1.32	0.003
Hypertension, n (%)	4137 (34.14%)	994 (33.01%)	969 (31.83%)	1057 (35.03%)	1117 (36.68%)	<0.001
History of myocardial infarction, n (%)	85 (0.70%)	27 (0.90%)	12 (0.39%)	18 (0.60%)	28 (0.92%)	0.040
History of stroke, n (%)	212 (1.75%)	44 (1.46%)	37 (1.22%)	67 (2.22%)	64 (2.10%)	0.006
ACEIs or ARBs, n (%)	296 (2.44%)	57 (1.89%)	66 (2.17%)	73 (2.42%)	100 (3.28%)	0.003
Beta blockers, n (%)	59 (0.49%)	5 (0.17%)	14 (0.46%)	15 (0.50%)	25 (0.82%)	0.004
Calcium channel blockers, n (%)	773 (6.38%)	127 (4.22%)	177 (5.81%)	211 (6.99%)	258 (8.47%)	<0.001
Diuretics, n (%)	17 (0.14%)	0 (0.00%)	3 (0.10%)	6 (0.20%)	8 (0.26%)	0.035
Other agents, n (%)	120 (0.99%)	21 (0.70%)	28 (0.92%)	34 (1.13%)	37 (1.22%)	0.177

*Abbreviations: BMR, basal metabolic rate; BMI, body mass index; SBP, systolic blood pressure; DBP, diastolic blood pressure; ACEIs, angiotensin-converting enzyme inhibitors; ARBs, angiotensin receptor blockers.*

Furthermore, the clinical characteristics of participants grouped by age or sex were also presented in Supplementary Table 2. The proportion of males and urban residence, prevalence of HT, history of myocardial infarction and stroke, and medication use (antihypertensive medications, ACEIs or ARBs, beta-blockers, and calcium channel blockers) were higher in elderly individuals (age ≥ 60 years) than in non-elderly subjects (*P* < 0.05). There was a higher prevalence of current smokers and drinkers as well as history of stroke, and higher values of SBP and DBP in male individuals compared with female subjects (*P* < 0.05).

### Association Between Age and the Basal Metabolic Rate

Considering that the BMR declines with age and the rate of decline accelerates at older ages (18), we used the generalized additive model and penalized spline method to assess the relationship between age and BMR. In the adjusted smoothing curve, the relationship between age and BMR was not linear. With the increase of age, the BMR fell slowly first and dropped significantly then subsequently (Supplementary Figure 2). Visual inspection showed that the inflection point was around 60 years old. We further fitted the association between age and BMR using the two-piecewise binary logistic regression model and the inflection point of age was 61 years old (Supplementary Table 3). Effect size [β(95% CI)] of age on BMR was –0.71 (–1.20, –0.22) on the left side and –5.21 (–5.77, –4.65) on the right side of the inflection point. These results suggested that there was a threshold effect of age on BMR.

### Cumulative Incidence of Death From All-Causes

For all participants, the mean follow-up time was 5.60 years. During this period, there were 809 deaths (6.68%), which included 478 men and 331 women. The all-cause mortality varied substantially among the four BMR categories (*P* < 0.001). The numbers of deaths were 245, 197, 213, and 154, with rates of death from all-causes at 8.14, 6.47, 7.06, and 5.06%, according to different BMR categories, respectively (Table 2). There were no statistically significant differences in the cause of death according to different BMR categories (*P* = 0.318). All-cause mortality was higher in elderly individuals than in non-elderly individuals (11.48 vs. 2.04%, *P* < 0.001) and was higher in male subjects than in female subjects (9.84 vs. 4.56%, *P* < 0.001, Supplementary Table 2).

**TABLE 2:** All-cause mortality of the study population according to quartiles of BMR.

Characteristics	Total subjects	Quartiles of BMR (kcal/day)				*P*-value
		Q1 [787, 1115]	Q2 [1116, 1219]	Q3 [1220, 1367]	Q4 [1368, 1789]	
Median follow-up time, years	5.60 (5.29–5.73)	5.62 (5.32–5.73)	5.61 (5.29–5.74)	5.60 (5.28–5.73)	5.58 (5.28–5.68)	0.541
All-cause mortality, n (%)	809 (6.68%)	245 (8.14%)	197 (6.47%)	213 (7.06%)	154 (5.06%)	<0.001
Cause of death, n (%)						0.318
Stroke	130 (16.07%)	38 (15.51%)	37 (18.78%)	33 (15.49%)	22 (14.29%)	
Cardiovascular disease	242 (29.91%)	78 (31.84%)	58 (29.44%)	66 (30.99%)	40 (25.97%)	
Malignant tumor	72 (8.90%)	17 (6.94%)	17 (8.63%)	21 (9.86%)	17 (11.04%)	
Respiratory failure	111 (13.72%)	29 (11.84%)	25 (12.69%)	33 (15.49%)	24 (15.58%)	
Others	134 (16.56%)	36 (14.69%)	29 (14.72%)	34 (15.96%)	35 (22.73%)	
Unknown	120 (14.83%)	47 (19.18%)	31 (15.74%)	26 (12.21%)	16 (10.39%)	

*Abbreviations: BMR, basal metabolic rate*.

### Association Between the Basal Metabolic Rate and All-Cause Mortality

The multivariable analyses indicated that the BMR was inversely associated with all-cause mortality (adjusted-HR per SD increase in confounder model: 0.89, 95% CI: 0.81–0.98, *P* = 0.018, Table 3). Compared with BMR levels ≤ 1,115 kcal/day, the highest BMR quartiles had lower all-cause mortality in the confounder model (adjusted-HR: 0.74, 95% CI: 0.57–0.96, *P* = 0.021; *P* for trend = 0.013, Table 3).

**TABLE 3:** Hazard ratios of different BMR categories for all-cause mortality.

Variables	Event, n (%)	Crude model		Model I		Model II	
		*HR (95%CI)*	*P-value*	*HR (95%CI)*	*P-value*	*HR (95%CI)*	*P-value*
**BMR (kcal/day)**							
Per SD increase	809 (6.68%)	0.82 (0.76, 0.88)	<0.001	0.80 (0.74, 0.87)	<0.001	0.89 (0.81, 0.98)	0.018
**Quartiles of BMR**							
Q1 [787, 1115]	245 (8.14%)	*Ref*		*Ref*		*Ref*	
Q2 [1116, 1219]	197 (6.47%)	0.78 (0.65, 0.95)	0.012	0.90 (0.74, 1.09)	0.269	0.95 (0.78, 1.16)	0.591
Q3 [1220, 1367]	213 (7.06%)	0.87 (0.72, 1.04)	0.126	0.82 (0.67, 1.00)	0.055	0.93 (0.75, 1.14)	0.470
Q4 [1368, 1789]	154 (5.06%)	0.62 (0.51, 0.76)	<0.001	0.57 (0.45, 0.72)	<0.001	0.74 (0.57, 0.96)	0.021
P for trend		<0.001		<0.001		0.013	

*Abbreviations: BMR, basal metabolic rate; *Ref*, reference; *HR*, hazard ratio; *CI*, confidence interval; SD, standard deviation.*

*Model I adjusted for age and gender.*

*Model II adjusted for age, sex, SBP, DBP, BMI, education level, current smokers and drinkers, physical activity levels, sleep duration on workdays or non-workdays, history of stroke, diuretics and calcium channel blockers usage.*

Then, we found that this association between BMR levels and all-cause mortality was modified by age and sex. There was a significant inverse relationship between BMR levels and all-cause mortality in elderly male individuals (adjusted-HR per SD increase: 0.80, 95% CI: 0.70–0.91, *P* < 0.001, Table 4). Compared with BMR levels ≤ 1,115 kcal/day, there was lower all-cause mortality in the third and highest BMR quartiles in elderly male subjects (adjusted-HR: 0.71, 95% CI: 0.53–0.95, *P* = 0.022; adjusted-HR: 0.60, 95% CI: 0.43–0.84, *P* = 0.003, respectively). Whether excluded or not of subjects with a history of myocardial infarction and stroke (Supplementary Table 4) or less than 1 year of follow-up (Supplementary Table 5), the hazard ratio and 95% CI were consistent. There was an inverse relationship between the BMR and all-cause mortality in elderly male individuals. Compared with BMR levels ≤ 1,115 kcal/day, there was lower all-cause mortality in the third and highest BMR quartiles in elderly male subjects.

**TABLE 4:** Hazard ratios of different BMR categories for all-cause mortality grouped by age and sex.

Variables	Event, n (%)	Crude model		Model I		Model II	
		HR (95%CI)	*P*-value	HR (95%CI)	*P*-value	HR (95%CI)	*P*-value
**Male**							

**Age < 60 years**							
**BMR (kcal/day)**							
Per SD increase	71 (3.15%)	0.83 (0.66, 1.05)	0.114	0.83 (0.66, 1.05)	0.121	0.95 (0.73, 1.24)	0.713
**Quartiles of BMR**							
Q1 [843, 1112]	3 (2.11%)	*Ref*		*Ref*		*Ref*	
Q2 [1120, 1219]	12 (5.22%)	2.38 (0.67, 8.42)	0.180	2.33 (0.66, 8.25)	0.191	2.23 (0.62, 8.04)	0.222
Q3 [1220, 1367]	20 (4.77%)	2.54 (0.75, 8.55)	0.133	2.20 (0.65, 7.41)	0.206	2.36 (0.69, 8.08)	0.170
Q4 [1368, 1789]	36 (2.46%)	1.36 (0.42, 4.41)	0.613	1.25 (0.38, 4.05)	0.716	1.55 (0.47, 5.13)	0.474
P for trend		0.193		0.143		0.634	
**Age ≥ 60 years**							
**BMR (kcal/day)**							
Per SD increase	407 (15.65%)	0.62 (0.56, 0.69)	<0.001	0.74 (0.66, 0.83)	<0.001	0.80 (0.70, 0.91)	<0.001
**Quartiles of BMR**							
Q1 [848, 1115]	75 (27.68%)	*Ref*		*Ref*		*Ref*	
Q2 [1116, 1218]	98 (20.42%)	0.69 (0.51, 0.93)	0.015	0.79 (0.58, 1.07)	0.124	0.78 (0.58, 1.07)	0.122
Q3 [1220, 1367]	129 (16.02%)	0.53 (0.40, 0.71)	<0.001	0.68 (0.51, 0.90)	0.008	0.71 (0.53, 0.95)	0.022
Q4 [1368, 1788]	105 (10.05%)	0.32 (0.24, 0.43)	<0.001	0.49 (0.36, 0.66)	<0.001	0.60 (0.43, 0.84)	0.003
P for trend		<0.001		<0.001		0.004	

**Female**							

**Age < 60 years**							
**BMR (kcal/day)**							
Per SD increase	55 (1.41%)	0.84 (0.56, 1.28)	0.423	0.84 (0.56, 1.27)	0.411	0.91 (0.55, 1.51)	0.723
**Quartiles of BMR**							
Q1 [797, 1115]	15 (1.41%)	*Ref*		*Ref*		*Ref*	
Q2 [1116, 1219]	21 (1.53%)	1.05 (0.54, 2.04)	0.880	1.04 (0.54, 2.03)	0.898	1.22 (0.61, 2.44)	0.571
Q3 [1220, 1367]	16 (1.41%)	0.99 (0.49, 1.99)	0.967	0.97 (0.48, 1.97)	0.943	1.10 (0.49, 2.49)	0.811
Q4 [1368, 1771]	3 (0.85%)	0.58 (0.17, 2.01)	0.394	0.57 (0.16, 1.96)	0.369	0.70 (0.17, 2.86)	0.624
P for trend		0.473		0.441		0.755	
**Age ≥ 60 years**							
**BMR (kcal/day)**							
Per SD increase	55 (1.41%)	0.74 (0.63, 0.87)	<0.001	0.97 (0.83, 1.13)	0.712	1.05 (0.89, 1.25)	0.550
**Quartiles of BMR**							
Q1 [787, 1115]	152 (9.89%)	*Ref*		*Ref*		*Ref*	
Q2 [1116, 1219]	66 (6.84%)	0.68 (0.51, 0.91)	0.010	0.92 (0.69, 1.24)	0.600	0.97 (0.71, 1.33)	0.844
Q3 [1220, 1367]	48 (7.25%)	0.72 (0.52, 1.00)	0.052	1.06 (0.76, 1.48)	0.740	1.23 (0.85, 1.79)	0.268
Q4 [1368, 1761]	10 (5.46%)	0.54 (0.28, 1.02)	0.058	0.71 (0.37, 1.34)	0.287	0.81 (0.42, 1.56)	0.525
P for trend		0.006		0.531		0.911	

*Abbreviations: BMR, basal metabolic rate; Ref, reference; HR, hazard ratio; CI, confidence interval; SD, standard deviation.*

*Model I adjusted for age.*

*Model II adjusted for age, SBP, DBP, BMI, education level, current smokers and drinkers, history of stroke and physical activity levels.*

**FIGURE 1:**
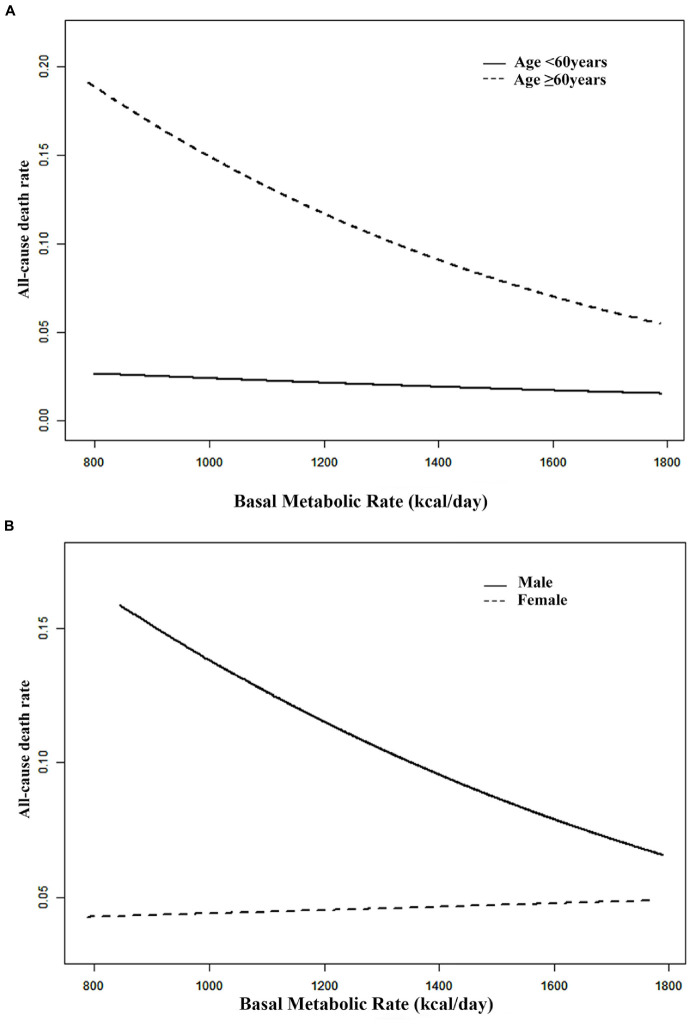
Smooth curve **(A)** adjusted for gender, SBP, DBP, BMI, education level, current smokers, physical activity levels and history of stroke. Smooth curve **(B)** adjusted for age, SBP, DBP, BMI, education level, current smokers and drinkers, history of stroke and physical activity levels.

**FIGURE 2:**
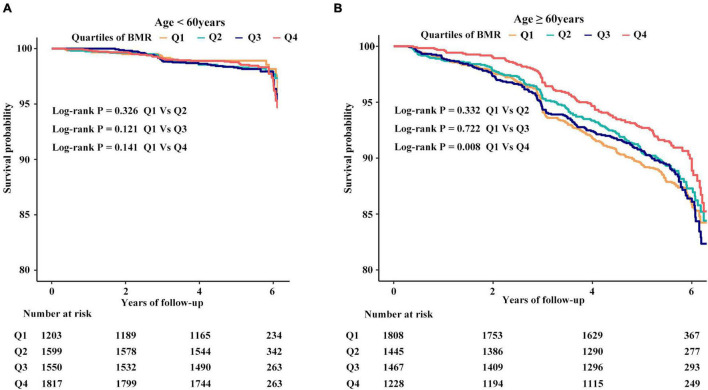
The cumulative hazards of all-cause mortality according to quartiles of BMR grouped by age. **(A)** The cumulative hazards of all-cause mortality in subjects with age < #60 years. **(B)** The cumulative hazards of all-cause mortality in subjects with age ≥ #60 years.

**FIGURE 3:**
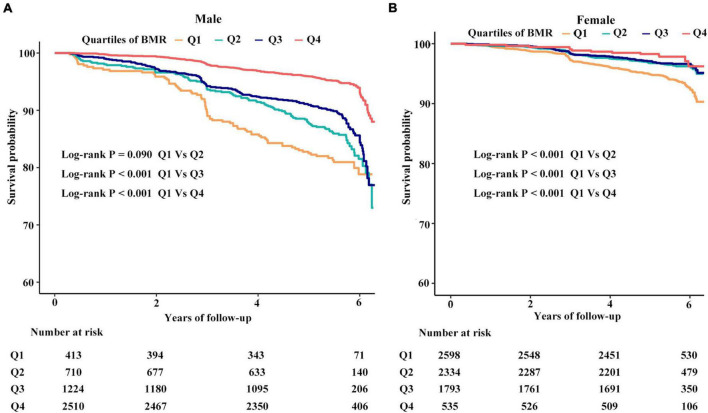
The cumulative hazards of all-cause mortality according to quartiles of BMR grouped by sex. **(A)** The cumulative hazards of all-cause mortality in males. **(B)** The cumulative hazards of all-cause mortality in females.

In the adjusted smoothing curve, the relationship between BMR levels and all-cause mortality was modified by age and sex (Figure 1). The survival analysis showed that compared with BMR levels ≤ 1,115 kcal/day, there was lower all-cause mortality in the highest BMR quartiles in elderly individuals (Kaplan-Meier, log-rank *P* = 0.141 or *P* = 0.008 for the highest BMR quartiles relative to the lowest BMR quartiles in the non-elderly or elderly population, respectively; Figure 2). Survival analysis found that compared with BMR levels ≤ 1,115 kcal/day, there was lower all-cause mortality in the highest BMR quartiles (Kaplan-Meier, log-rank *P* < 0.001 or *P* < 0.001 for the highest BMR quartiles relative to the lowest BMR quartiles in female or male subjects, respectively; Figure 3).

### Subgroup Analyses by Potential Effect Modifiers

To explore whether this association between the BMR and all-cause mortality was still stable in different subgroups, we conducted the stratified and interaction analyses. The subgroup analyses showed that there were no significant interactions between different BMR categories and all-cause mortality in any of the subgroups, including education level (primary school or below vs. middle school or above), SBP dichotomy (low vs. high), current smokers (no vs. yes), current drinkers (no vs. yes), and usage of cardiovascular drugs (no vs. yes) (Supplementary Figure 3).

It is noted that the composition of the BMI group across BMR quartiles differs. Besides, BMI is closely associated with BMR. We found that the relationship between the BMR and all-cause mortality was modified by BMI levels. There was a significantly inverse relationship between the BMR and all-cause mortality only in the overweight or general obesity subgroup (adjusted-HR per SD increase in the overweight subgroup: 0.80, 95% CI: 0.66–0.98, *P* = 0.027, adjusted-HR per SD increase in general obesity subgroup: 0.68, 95% CI: 0.48–0.97, *P* = 0.033; Supplementary Table 6). Stratified analyses also found that there was an inverse relationship between the BMR and all-cause mortality only in subjects with age ≥ 65 years [adjusted-HR per SD increase in the subgroup with age (65, 74) years: 0.80, 95% CI: 0.67–0.94, *P* = 0.007, adjusted-HR per SD increase in the subgroup with age ≥ 75 years: 0.81, 95% CI: 0.71–0.92, *P* = 0.001; Supplementary Table 7].

## Discussion

On the basis of data collected longitudinally from healthy community-dwelling persons who participated in the National Key R&D Program, we found that BMR declined non-linearly with age. We confirmed that BMR declines with age, and the rate of decline accelerates at older ages (Rizzo et al., 2005). Interestingly, the relationship based on longitudinal data between BMR and all-cause mortality was modified by age and sex. Accordingly, an elevated BMR was independently inversely associated with all-cause mortality in elderly male subjects in a southern Chinese adult population.

In particular, this finding was the first study showing that reduced BMR is a significant risk factor for mortality in the elderly population. This result challenged the previous concept that people who live longer are able to preserve a low energy metabolism (Ruggiero et al., 2008; Jumpertz et al., 2011; Schrack et al., 2014). In reality, the sample of the BLSA study mostly included a highly educated white population of high socioeconomic status and women who were enrolled only from 1978 (Data were collected from 1958 through 1982). In addition, BMR was estimated from basal oxygen (O_2_) consumption and carbon dioxide (CO_2)_ production using the open-circuit method using a Haldane apparatus until 1965. After then, they transformed to use a paramagnetic O_2_ analyzer and infrared absorption CO_2_ analyzer. After the two analytical systems were shown to be equivalent, all subsequent analyses were performed by the more modern method. BMR was calculated from respiratory data using Lusk’s tables based on DuBois’s equation to estimate the body surface area (Ruggiero et al., 2008; Schrack et al., 2014). In another study that enrolled non-diabetic healthy Pima Indian volunteers, BMR was measured using an open-circuit respiratory hood system (Jumpertz et al., 2011).

In our Chinese cohort, the BMR was measured using an Omron body fat analyzer (Ali et al., 2017). These differences in ethnicity or methods in measuring the BMR might contribute to the discrepancy of these outcomes.

In fact, the probability of survival dropped markedly in individuals over the age of 80 years, and the mortality rate increased exponentially up to the age of 100 years (Barbieri et al., 2005). The renormalized msBMR (RmsBMR), which was derived in one cohort of American men (*n* = 25,425) by incorporating the body mass index into the Harris-Benedict equation, was identified as one of the best biomarkers of aging on the mortality rate and survival curve (Barbieri et al., 2005). Particularly, the plateau of the mortality rate with age in centenarians was corresponded to the lowest limit (threshold) of the renormalized mass-specific BMR (RmsBMR), which stands for the final stage of human life (Barbi et al., 2018). The plateau effect prolonged the longevity especially without exceeding the age of 120 years. A general decrease of the RmsBMR with age was associated with the mitochondrial number decay, which was caused by a slight fluctuation of the dynamic fusion/fission system (Kitazoe et al., 2019). Some organs and tissues are likely to fall into a state of dysfunction in elderly individuals since the cellular energy supply gradually becomes insufficient such that diseases manifest, which may cause the death of these individuals (Kitazoe et al., 2019). In our study, insufficient physical energy supply in elderly individuals may explain why a lower BMR was significantly associated with an increased risk of all-cause mortality. There was a higher prevalence of current smokers and drinkers as well as a history of stroke in male individuals than in female individuals, which may explain why the relationship of the BMR with all-cause mortality was modified by sex.

It was well established that the health benefits of chronic caloric restriction (CR) on lifespan extension might be traced to lowering of BMR in many species and non-human primates. However, after a transient initial response, CR did not always result in reduced BMR in the long run (McCarter et al., 1985; McCarter and McGee, 1989; Ramsey and Hagopian, 2006; Raman et al., 2007; Pifferi and Aujard, 2019). In addition, experimentally elevated energy expenditure in rodents, such as increasing the amount of voluntary aerobic exercise or lifelong cold exposure, did not lead to anticipated reductions in lifespan (Selman et al., 2008; Vaanholt et al., 2010).

It is worth noting that the effect of BMR on lifespan was usually related to other components of energy metabolism. The longevous mice not only had elevated BMR but also raised total daily energy expenditures and elevated expenditure on physical activity (Speakman et al., 2004). The “uncoupled and surviving” hypothesis proposed that the increased mitochondrial proton cycling leads to oxidation of ubiquinone and decreased ROS production consistent with increased lifespan (Brand, 2000). It is just as likely that the longevity of these mice was owing to their raised expenditure on physical activity (Speakman et al., 2004).

Several limitations of our study should be addressed in interpreting the results. First, considering the seasonal rhythm for BMR (Didikoglu et al., 2020), our research lacked dynamic and longitudinal BMR evaluation. Second, the length of follow-up of the participants was relatively short compared with the BLSA (Ruggiero et al., 2008). In addition, the equation estimation for BMR was simple and convenient, which is suitable for large samples, but lowered the accuracy of measurement relative to indirect calorimetry (Zhang et al., 2018).

## Conclusion

An elevated BMR was independently inversely associated with all-cause mortality in elderly male subjects in a southern Chinese population. Our findings may make a contribution to a new prospective study to investigate the effect of BMR on all-cause mortality and human longevity. Future research is needed to explore optimized energy utilization and regulation for the sake of the compression of morbidity and promotion of longevity.

## Data Availability Statement

The raw data supporting the conclusions of this article will be made available by the authors, without undue reservation.

## Ethics Statement

The studies involving human participants were reviewed and approved by the Second Affiliated Hospital of Nanchang University and the Fuwai Cardiovascular Hospital. The patients/participants provided their written informed consent to participate in this study.

## Author Contributions

FYH and FH: conception and design, data acquisition and analysis, interpretation, drafting, final approval, and contributed to this study equally. TW and WZ: design and final approval. LZ and XH: analysis and interpretation and final approval. HB and XC: conception and design, critical revision, and final approval. All authors contributed to the article and approved the submitted version.

## Conflict of Interest

The authors declare that the research was conducted in the absence of any commercial or financial relationships that could be construed as a potential conflict of interest.

## Publisher’s Note

All claims expressed in this article are solely those of the authors and do not necessarily represent those of their affiliated organizations, or those of the publisher, the editors and the reviewers. Any product that may be evaluated in this article, or claim that may be made by its manufacturer, is not guaranteed or endorsed by the publisher.

## Footnotes

1
http://www.R-project.org

## Supplementary Material

Supplementary Figure 1 Flowchart of this analysis.

Supplementary Figure 2 Smooth curve of correlations between age and basal metabolic rate.

Supplementary Figure 3 Effect size of quartiles of basal metabolic rate on all-cause mortality in prespecified and exploratory subgroups. Note: Each stratification adjusted for age, gender, SBP, DBP, BMI, education level, current smokers and drinkers, physical activity levels, sleep duration on workdays or non-workdays, history of stroke, diuretics, and calcium channel blocker usage except the subgroup variable. The usage of cardiovascular drugs included ACEI or ARB, beta-blockers, calcium channel blockers, and diuretics. Ref, reference; HR, hazard ratio; CI, confidence interval; ACEI, angiotensin-converting enzyme inhibitor; ARB, angiotensin receptor blocker.
