# Corrigendum: Association between basal metabolic rate and all-cause mortality in a prospective cohort of southern Chinese adults

**DOI:** 10.3389/fphys.2025.1594299

**Published:** 2025-04-17

**Authors:** Fengyu Han, Feng Hu, Tao Wang, Wei Zhou, Linjuan Zhu, Xiao Huang, Huihui Bao, Xiaoshu Cheng

**Affiliations:** ^1^ The Department of Cardiovascular Medicine, The Second Affiliated Hospital of Nanchang University, Nanchang, China; ^2^ Jiangxi Provincial Cardiovascular Disease Clinical Medical Research Center, Nanchang, China; ^3^ Center for Prevention and Treatment of Cardiovascular Diseases, The Second Affiliated Hospital of Nanchang University, Nanchang, China

**Keywords:** basal metabolic rate, all-cause mortality, Chinese, adults, aging

In the published article, there was an error in Table 1–4 and Figure 1 as published. The incorrect unit for BMR was mistakenly used. The correct unit for BMR should be kcal/day, instead of kJ/day. The corrected Table 1–4 and Figure 1 and their captions appear below.

**TABLE 1:** Baseline characteristics of the study population according to quartiles of BMR.

Characteristics	Total subjects	Quartiles of BMR (kcal/day)				*P-value*
		Q1 [787, 1115]	Q2 [1116, 1219]	Q3 [1220, 1367]	Q4 [1368, 1789]	
Number of subjects (n)	12,117	3011	3044	3017	3045	
Age (years)	59.04 ± 13.21	62.53 ± 14.16	58.54 ± 13.05	58.79 ± 12.64	56.35 ± 12.19	<0.001
Male, n (%)	4857 (40.08%)	413 (13.72%)	710 (23.32%)	1224 (40.57%)	2510 (82.43%)	<0.001
SBP (mmHg)	127.95 ± 19.62	127.45 ± 21.02	126.19 ± 19.66	128.11 ± 19.37	130.04 ± 18.17	<0.001
DBP (mmHg)	74.80 ± 10.75	72.47 ± 10.63	73.75 ± 10.35	75.24 ± 10.43	77.70 ± 10.85	<0.001
BMR (kcal/day)	1251.79 ± 190.02	1034.34 ± 66.90	1166.52 ± 31.27	1288.77 ± 43.34	1515.40 ± 107.41	<0.001
BMI (kg/m^2^)	23.07 ± 3.65	21.04 ± 2.91	22.64 ± 2.86	23.61 ± 3.56	24.95 ± 3.97	<0.001
BMI group (kg/m^^2^)						<0.001
Underweight (<18.5)	904 (7.47%)	484 (16.02%)	195 (6.41%)	164 (5.44%)	63 (2.07%)	
Normal weight (≥18.5, <24)	6787 (56.05%)	2158 (71.72%)	1974 (64.91%)	1449 (48.06%)	1206 (39.62%)	
Overweight (≥24, <28)	3439 (28.40%)	319 (10.60%)	786 (25.85%)	1104 (36.62%)	1230 (40.41%)	
General obesity (≥28)	979 (8.08%)	50 (1.66%)	86 (2.83%)	298 (9.88%)	545 (17.90%)	
Waist circumference (cm)	79.90 ± 9.14	74.14 ± 7.69	78.15 ± 7.40	81.07 ± 8.11	86.19 ± 8.80	<0.001
Urban residence, n (%)	6199 (51.16%)	1392 (46.23%)	1543 (50.69%)	1611 (53.40%)	1653 (54.29%)	<0.001
Education level, n (%)						<0.001
Primary school or below	7002 (58.51%)	2189 (74.15%)	1861 (61.81%)	1795 (60.40%)	1157 (38.16%)	
Middle school	4577 (38.25%)	727 (24.63%)	1082 (35.93%)	1108 (37.28%)	1660 (54.75%)	
Graduate and above	388 (3.24%)	36 (1.22%)	68 (2.26%)	69 (2.32%)	215 (7.09%)	
Current smokers, n (%)	2338 (19.34%)	219 (7.28%)	358 (11.78%)	623 (20.73%)	1138 (37.50%)	<0.001
Current drinkers, n (%)	3015 (24.96%)	487 (16.23%)	595 (19.60%)	718 (23.89%)	1215 (40.03%)	<0.001
Physical activity levels, n (%)						<0.001
Low	1832 (15.12%)	470 (15.61%)	407 (13.37%)	410 (13.59%)	545 (17.90%)	
Middle	3208 (26.48%)	847 (28.13%)	811 (26.64%)	764 (25.32%)	786 (25.81%)	
High	7047 (58.16%)	1690 (56.13%)	1820 (59.79%)	1829 (60.62%)	1708 (56.09%)	
Sleep duration on workdays (hours)	7.29 ± 1.32	7.24 ± 1.38	7.33 ± 1.29	7.32 ± 1.30	7.26 ± 1.29	0.016
Sleep duration on non-workdays (hours)	7.56 ± 1.36	7.49 ± 1.43	7.61 ± 1.33	7.59 ± 1.35	7.55 ± 1.32	0.003
Hypertension, n (%)	4137 (34.14%)	994 (33.01%)	969 (31.83%)	1057 (35.03%)	1117 (36.68%)	<0.001
History of myocardial infarction, n (%)	85 (0.70%)	27 (0.90%)	12 (0.39%)	18 (0.60%)	28 (0.92%)	0.040
History of stroke, n (%)	212 (1.75%)	44 (1.46%)	37 (1.22%)	67 (2.22%)	64 (2.10%)	0.006
ACEIs or ARBs, n (%)	296 (2.44%)	57 (1.89%)	66 (2.17%)	73 (2.42%)	100 (3.28%)	0.003
Beta blockers, n (%)	59 (0.49%)	5 (0.17%)	14 (0.46%)	15 (0.50%)	25 (0.82%)	0.004
Calcium channel blockers, n (%)	773 (6.38%)	127 (4.22%)	177 (5.81%)	211 (6.99%)	258 (8.47%)	<0.001
Diuretics, n (%)	17 (0.14%)	0 (0.00%)	3 (0.10%)	6 (0.20%)	8 (0.26%)	0.035
Other agents, n (%)	120 (0.99%)	21 (0.70%)	28 (0.92%)	34 (1.13%)	37 (1.22%)	0.177

Abbreviations: BMR, basal metabolic rate; BMI, body mass index; SBP, systolic blood pressure; DBP, diastolic blood pressure; ACEIs, angiotensin-converting enzyme inhibitors; ARBs, angiotensin receptor blockers.

**TABLE 2:** All-cause mortality of the study population according to quartiles of BMR.

Characteristics	Total subjects	Quartiles of BMR (kcal/day)				*P-value*
		Q1 [787, 1115]	Q2 [1116, 1219]	Q3 [1220, 1367]	Q4 [1368, 1789]	
Median follow-up time, years	5.60 (5.29–5.73)	5.62 (5.32–5.73)	5.61 (5.29–5.74)	5.60 (5.28–5.73)	5.58 (5.28–5.68)	0.541
All-cause mortality, n (%)	809 (6.68%)	245 (8.14%)	197 (6.47%)	213 (7.06%)	154 (5.06%)	<0.001
Cause of death, n (%)						0.318
Stroke	130 (16.07%)	38 (15.51%)	37 (18.78%)	33 (15.49%)	22 (14.29%)	
Cardiovascular disease	242 (29.91%)	78 (31.84%)	58 (29.44%)	66 (30.99%)	40 (25.97%)	
Malignant tumor	72 (8.90%)	17 (6.94%)	17 (8.63%)	21 (9.86%)	17 (11.04%)	
Respiratory failure	111 (13.72%)	29 (11.84%)	25 (12.69%)	33 (15.49%)	24 (15.58%)	
Others	134 (16.56%)	36 (14.69%)	29 (14.72%)	34 (15.96%)	35 (22.73%)	
Unknown	120 (14.83%)	47 (19.18%)	31 (15.74%)	26 (12.21%)	16 (10.39%)	

Abbreviations: BMR, basal metabolic rate.

**TABLE 3:** Hazard ratios of different BMR categories for all-cause mortality.

Variables	Event, n (%)	Crude model		Model Ⅰ		Model Ⅱ	
		*HR (95%CI)*	*P-value*	*HR (95%CI)*	*P-value*	*HR (95%CI)*	*P-value*
BMR (kcal/day)							
Per SD increase	809 (6.68%)	0.82 (0.76, 0.88)	<0.001	0.80 (0.74, 0.87)	<0.001	0.89 (0.81, 0.98)	0.018
Quartiles of BMR							
Q1 [787, 1115]	245 (8.14%)	*Ref*		*Ref*		*Ref*	
Q2 [1116, 1219]	197 (6.47%)	0.78 (0.65, 0.95)	0.012	0.90 (0.74, 1.09)	0.269	0.95 (0.78, 1.16)	0.591
Q3 [1220, 1367]	213 (7.06%)	0.87 (0.72, 1.04)	0.126	0.82 (0.67, 1.00)	0.055	0.93 (0.75, 1.14)	0.470
Q4 [1368, 1789]	154 (5.06%)	0.62 (0.51, 0.76)	<0.001	0.57 (0.45, 0.72)	<0.001	0.74 (0.57, 0.96)	0.021
P for trend		<0.001		<0.001		0.013	

Abbreviations: BMR, basal metabolic rate; *Ref*, reference; *HR*, hazard ratio; *CI*, confidence interval; SD, standard deviation.

Model Ⅰ adjusted for age and gender.

Model Ⅱ adjusted for age, sex, SBP, DBP, BMI, education level, current smokers and drinkers, physical activity levels, sleep duration on workdays or non-workdays, history of stroke, diuretics and calcium channel blockers usage.

**TABLE 4:** Hazard ratios of different BMR categories for all-cause mortality grouped by age and sex.

Variables	Event, n (%)	Crude model		Model Ⅰ		Model Ⅱ	
		HR (95%CI)	P-value	HR (95%CI)	P-value	HR (95%CI)	P-value
Male							
Age <60years							
BMR (kcal/day)							
Per SD increase	71 (3.15%)	0.83 (0.66, 1.05)	0.114	0.83 (0.66, 1.05)	0.121	0.95 (0.73, 1.24)	0.713
Quartiles of BMR							
Q1 [843, 1112]	3 (2.11%)	*Ref*		*Ref*		*Ref*	
Q2 [1120, 1219]	12 (5.22%)	2.38 (0.67, 8.42)	0.180	2.33 (0.66, 8.25)	0.191	2.23 (0.62, 8.04)	0.222
Q3 [1220, 1367]	20 (4.77%)	2.54 (0.75, 8.55)	0.133	2.20 (0.65, 7.41)	0.206	2.36 (0.69, 8.08)	0.170
Q4 [1368, 1789]	36 (2.46%)	1.36 (0.42, 4.41)	0.613	1.25 (0.38, 4.05)	0.716	1.55 (0.47, 5.13)	0.474
P for trend		0.193		0.143		0.634	
Age ≥60years							
BMR (kcal/day)							
Per SD increase	407 (15.65%)	0.62 (0.56, 0.69)	<0.001	0.74 (0.66, 0.83)	<0.001	0.80 (0.70, 0.91)	<0.001
Quartiles of BMR							
Q1 [848, 1115]	75 (27.68%)	*Ref*		*Ref*		*Ref*	
Q2 [1116, 1218]	98 (20.42%)	0.69 (0.51, 0.93)	0.015	0.79 (0.58, 1.07)	0.124	0.78 (0.58, 1.07)	0.122
Q3 [1220, 1367]	129 (16.02%)	0.53 (0.40, 0.71)	<0.001	0.68 (0.51, 0.90)	0.008	0.71 (0.53, 0.95)	0.022
Q4 [1368, 1788]	105 (10.05%)	0.32 (0.24, 0.43)	<0.001	0.49 (0.36, 0.66)	<0.001	0.60 (0.43, 0.84)	0.003
P for trend		<0.001		<0.001		0.004	
Female							
Age <60years							
BMR (kcal/day)							
Per SD increase	55 (1.41%)	0.84 (0.56, 1.28)	0.423	0.84 (0.56, 1.27)	0.411	0.91 (0.55, 1.51)	0.723
Quartiles of BMR							
Q1 [797, 1115]	15 (1.41%)	*Ref*		*Ref*		*Ref*	
Q2 [1116, 1219]	21 (1.53%)	1.05 (0.54, 2.04)	0.880	1.04 (0.54, 2.03)	0.898	1.22 (0.61, 2.44)	0.571
Q3 [1220, 1367]	16 (1.41%)	0.99 (0.49, 1.99)	0.967	0.97 (0.48, 1.97)	0.943	1.10 (0.49, 2.49)	0.811
Q4 [1368, 1771]	3 (0.85%)	0.58 (0.17, 2.01)	0.394	0.57 (0.16, 1.96)	0.369	0.70 (0.17, 2.86)	0.624
P for trend		0.473		0.441		0.755	
Age ≥60years							
BMR (kcal/day)							
Per SD increase	55 (1.41%)	0.74 (0.63, 0.87)	<0.001	0.97 (0.83, 1.13)	0.712	1.05 (0.89, 1.25)	0.550
Quartiles of BMR							
Q1 [787, 1115]	152 (9.89%)	*Ref*		*Ref*		*Ref*	
Q2 [1116, 1219]	66 (6.84%)	0.68 (0.51, 0.91)	0.010	0.92 (0.69, 1.24)	0.600	0.97 (0.71, 1.33)	0.844
Q3 [1220, 1367]	48 (7.25%)	0.72 (0.52, 1.00)	0.052	1.06 (0.76, 1.48)	0.740	1.23 (0.85, 1.79)	0.268
Q4 [1368, 1761]	10 (5.46%)	0.54 (0.28, 1.02)	0.058	0.71 (0.37, 1.34)	0.287	0.81 (0.42, 1.56)	0.525
P for trend		0.006		0.531		0.911	

Abbreviations: BMR, basal metabolic rate; Ref, reference; HR, hazard ratio; CI, confidence interval; SD, standard deviation.

Model Ⅰ adjusted for age.

Model Ⅱ adjusted for age, SBP, DBP, BMI, education level, current smokers and drinkers, history of stroke and physical activity levels.

**FIGURE 1:**
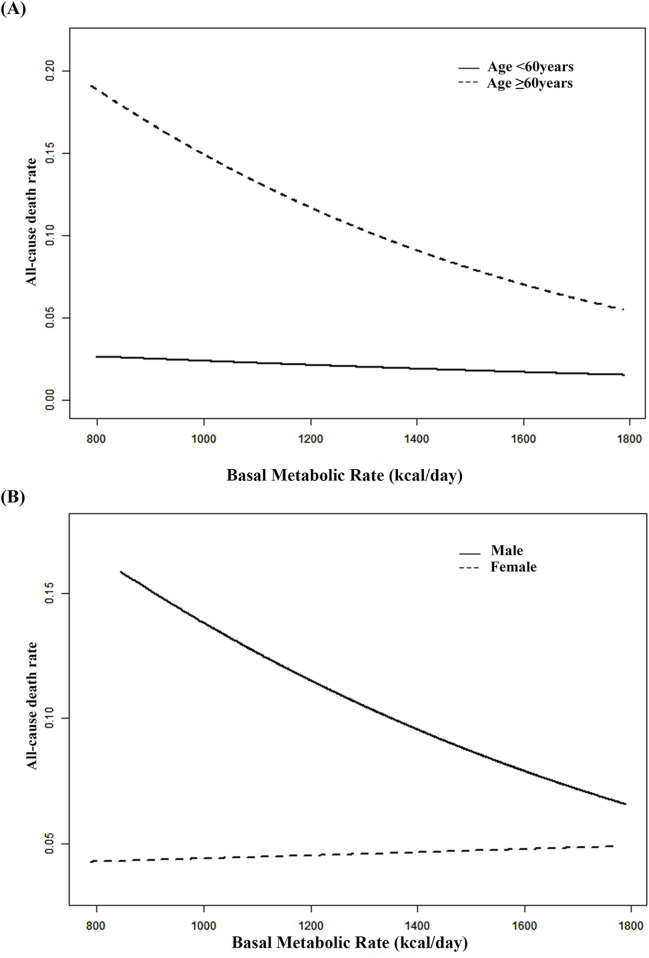
Smooth curve **(A)** adjusted for gender, SBP, DBP, BMI, education level, current smokers, physical activity levels and history of stroke. Smooth curve **(B)** adjusted for age, SBP, DBP, BMI, education level, current smokers and drinkers, history of stroke and physical activity levels.

In the published article, there was an error in **Supplementary Figure 2**, **Supplementary Tables 2, 4, 5**. The incorrect unit for BMR was mistakenly used. The correct unit for BMR should be kcal/day, instead of kJ/day. The correct supplementary material has been published with the original article.

In the published article, there was an error. The incorrect unit for BMR was mistakenly used. The correct unit for BMR should be kcal/day, instead of kJ/day.

A correction has been made to **Abstract**, Result. This sentence previously stated:

“There was a significantly inverse relationship between BMR levels and all-cause mortality in elderly male individuals (adjusted-HR per SD increase: 0.80, 95% CI: 0.70–0.91, P < 0.001). Compared with BMR levels ≤ 1,115 kJ/day, there was lower all-cause mortality in third and highest BMR quartiles in the elderly male subjects (adjusted-HR: 0.71, 95% CI:0.53–0.95, P = 0.022; adjusted-HR: 0.60, 95% CI: 0.43–0.84, P = 0.003, respectively.”

The corrected sentence appears below:

“There was a significantly inverse relationship between BMR levels and all-cause mortality in elderly male individuals (adjusted-HR per SD increase: 0.80, 95% CI: 0.70–0.91, P < 0.001). Compared with BMR levels ≤ 1,115 kcal/day, there was lower all-cause mortality in third and highest BMR quartiles in the elderly male subjects (adjusted-HR: 0.71, 95% CI:0.53–0.95, P = 0.022; adjusted-HR: 0.60, 95% CI: 0.43–0.84, P = 0.003, respectively.”

A correction has been made to **Results**, Association Between the Basal Metabolic Rate and All-Cause Mortality, Paragraph 1. This sentence previously stated:

“The multivariable analyses indicated that the BMR was inversely associated with all-cause mortality (adjusted-HR per SD increase in confounder model: 0.89, 95% CI: 0.81–0.98, P = 0.018, Table 3). Compared with BMR levels ≤1,115 kJ/day, the highest BMR quartiles had lower all-cause mortality in the confounder model (adjusted-HR: 0.74, 95% CI: 0.57–0.96, P = 0.021; P for trend = 0.013, Table 3).”

The corrected sentence appears below:

“The multivariable analyses indicated that the BMR was inversely associated with all-cause mortality (adjusted-HR per SD increase in confounder model: 0.89, 95% CI: 0.81–0.98, P = 0.018, Table 3). Compared with BMR levels ≤1,115 kcal/day, the highest BMR quartiles had lower all-cause mortality in the confounder model (adjusted-HR: 0.74, 95% CI: 0.57–0.96, P = 0.021; P for trend = 0.013, Table 3).”

A correction has been made to **Results**, Association Between the Basal Metabolic Rate and All-Cause Mortality, Paragraph 2. This sentence previously stated:

“Compared with BMR levels ≤ 1,115 kJ/day, there was lower all-cause mortality in the third and highest BMR quartiles in elderly male subjects (adjusted-HR: 0.71, 95% CI: 0.53–0.95, P = 0.022; adjusted-HR: 0.60, 95% CI: 0.43–0.84, P = 0.003, respectively).”

The corrected sentence appears below:

“Compared with BMR levels ≤ 1,115 kcal/day, there was lower all-cause mortality in the third and highest BMR quartiles in elderly male subjects (adjusted-HR: 0.71, 95% CI: 0.53–0.95, P = 0.022; adjusted-HR: 0.60, 95% CI: 0.43–0.84, P = 0.003, respectively).”

A correction has been made to **Results**, Association Between the Basal Metabolic Rate and All-Cause Mortality, Paragraph 2. This sentence previously stated:

“There was an inverse relationship between the BMR and all-cause mortality in elderly male individuals. Compared with BMR levels ≤1,115 kJ/day, there was lower all-cause mortality in the third and highest BMR quartiles in elderly male subjects.”

The corrected sentence appears below:

“There was an inverse relationship between the BMR and all-cause mortality in elderly male individuals. Compared with BMR levels ≤1,115 kcal/day, there was lower all-cause mortality in the third and highest BMR quartiles in elderly male subjects.”

A correction has been made to **Results**, Association Between the Basal Metabolic Rate and All-Cause Mortality, Paragraph 3. This sentence previously stated:

“The survival analysis showed that compared with BMR levels ≤ 1,115 kJ/day, there was lower all-cause mortality in the highest BMR quartiles in elderly individuals (Kaplan-Meier, log-rank P = 0.141 or P = 0.008 for the highest BMR quartiles relative to the lowest BMR quartiles in the non-elderly or elderly population, respectively; Figure 2).”

The corrected sentence appears below:

“The survival analysis showed that compared with BMR levels ≤ 1,115 kcal/day, there was lower all-cause mortality in the highest BMR quartiles in elderly individuals (Kaplan-Meier, log-rank P = 0.141 or P = 0.008 for the highest BMR quartiles relative to the lowest BMR quartiles in the non-elderly or elderly population, respectively; Figure 2).”

A correction has been made to **Results**, Association Between the Basal Metabolic Rate and All-Cause Mortality, Paragraph 3. This sentence previously stated:

“Survival analysis found that compared with BMR levels ≤ 1,115 kJ/day, there was lower all-cause mortality in the highest BMR quartiles (Kaplan-Meier, log-rank P < 0.001 or P < 0.001 for the highest BMR quartiles relative to the lowest BMR quartiles in female or male subjects, respectively; Figure 3).”

The corrected sentence appears below:

“Survival analysis found that compared with BMR levels ≤ 1,115 kcal/day, there was lower all-cause mortality in the highest BMR quartiles (Kaplan-Meier, log-rank P < 0.001 or P < 0.001 for the highest BMR quartiles relative to the lowest BMR quartiles in female or male subjects, respectively; Figure 3).”

A correction has been made to **Discussion**, Paragraph 6. This sentence previously stated:

“The longevity mice not only had elevated BMR but also raised total daily energy expenditures and elevated expenditure on physical activity.”

The corrected sentence appears below:

“The longevous mice not only had elevated BMR but also raised total daily energy expenditures and elevated expenditure on physical activity.”

The authors apologize for these errors and state that this does not change the scientific conclusions of the article in any way. The original article has been updated.

