# Supplementary figures and images for: Association Between Basal Metabolic Rate and All-Cause Mortality in a Prospective Cohort of Southern Chinese Adults
Source: Front Physiol. 2022 Jan 4;12:790347. doi: 10.3389/fphys.2021.790347 (PMC8763786; doi:10.3389/fphys.2021.790347)

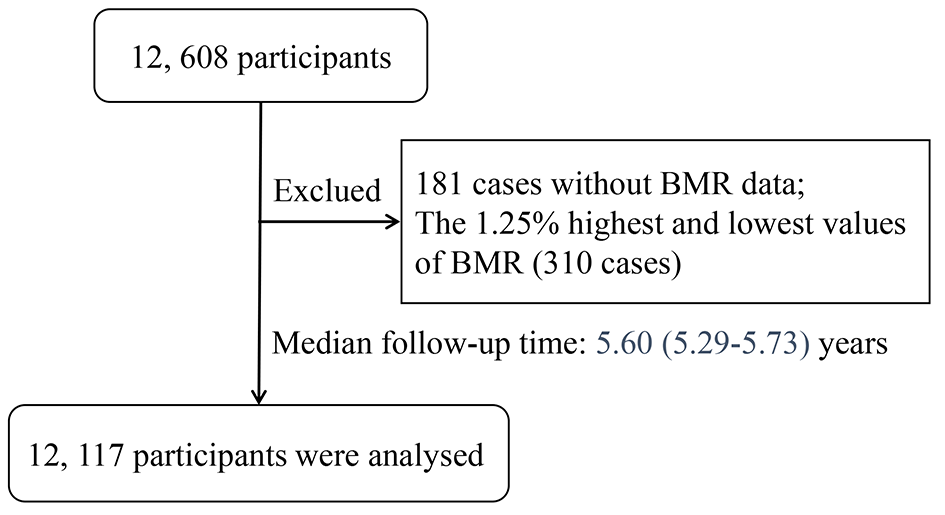

Supplement: Supplementary Figure 1 — Flowchart of this analysis. [file Image_1.TIF]

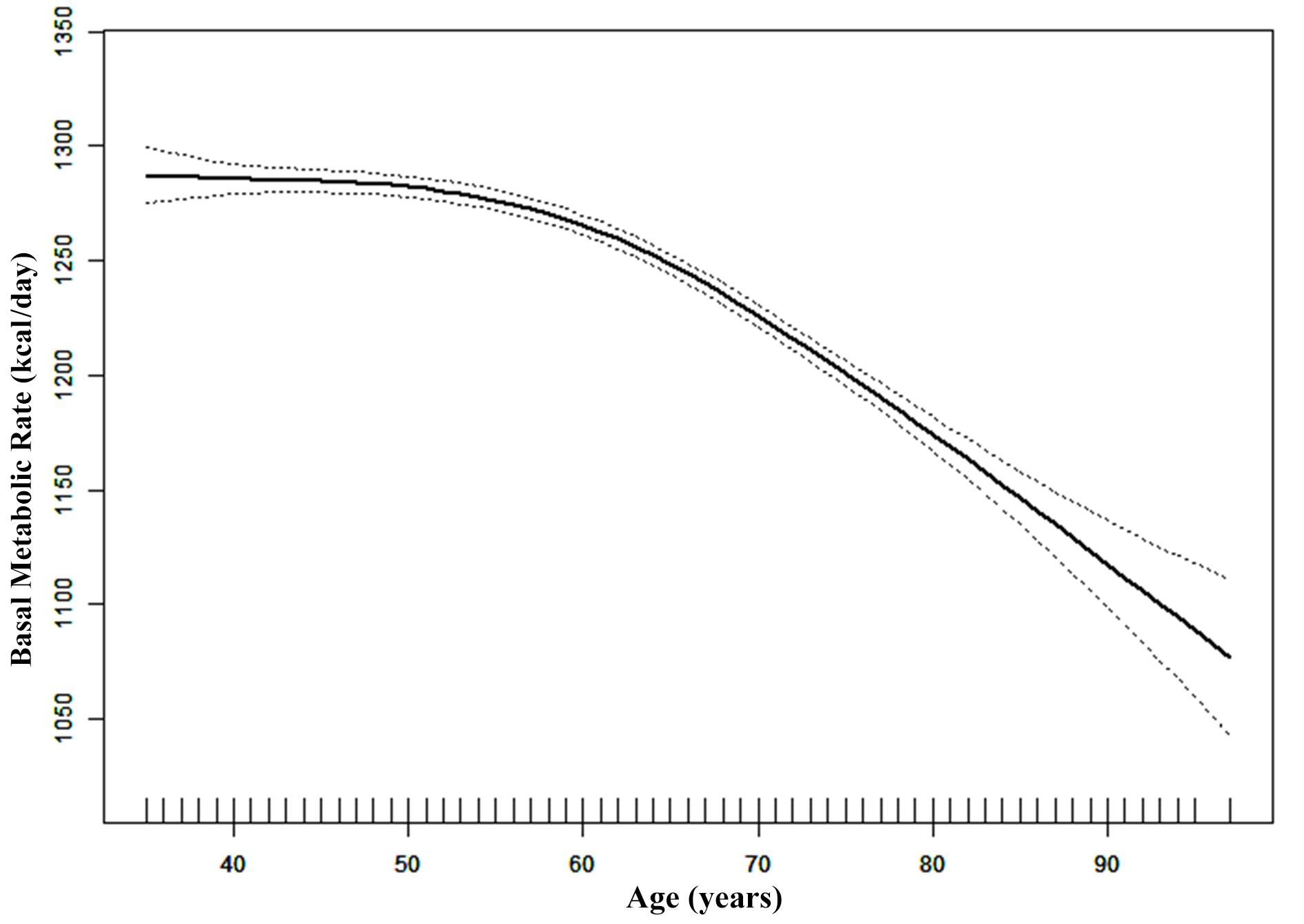

Supplement: Supplementary Figure 2 — Smooth curve of correlations between age and basal metabolic rate. [file Image_2.tif]

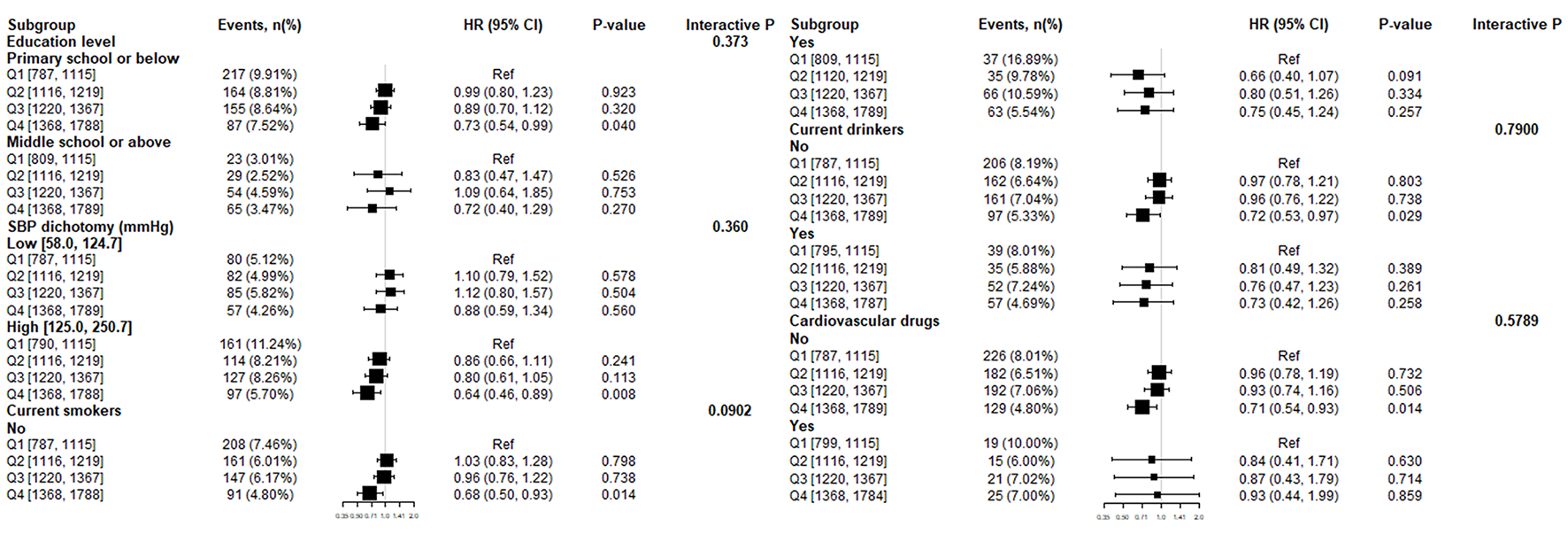

Supplement: Supplementary Figure 3 — Effect size of quartiles of basal metabolic rate on all-cause mortality in prespecified and exploratory subgroups. Note: Each stratification adjusted for age, gender, SBP, DBP, BMI, education level, current smokers and drinkers, physical activity levels, sleep duration on workdays or non-workdays, history of stroke, diuretics, and calcium channel blocker usage except the subgroup variable. The usage of cardiovascular drugs included ACEI or ARB, beta-blockers, calcium channel blockers, and diuretics. Ref, reference; HR, hazard ratio; CI, confidence interval; ACEI, angiotensin-converting enzyme inhibitor; ARB, angiotensin receptor blocker. [file Image_3.TIF]
