# Supplementary material for: Association Between Basal Metabolic Rate and All-Cause Mortality in a Prospective Cohort of Southern Chinese Adults
Source: Front Physiol. 2022 Jan 4;12:790347. doi: 10.3389/fphys.2021.790347 (PMC8763786; doi:10.3389/fphys.2021.790347)
Supplement: Supplementary file 4 [file Data_Sheet_1.docx]

**Supplementary Table legends**

| **Supplementary Table 1.** Associations of covariates with all-cause mortality. | | |
| --- | --- | --- |
| Covariates | All-cause mortality | |
|  | *HR (95%CI)* | *P-value* |
| Age (years) | 1.09 (1.08, 1.10) | <0.001 |
| Gender, n(%) |  |  |
| Male | *Ref* |  |
| Female | 0.44 (0.38, 0.51) | <0.001 |
| Education level, n(%) |  |  |
| Primary school or below | *Ref* |  |
| Middle school | 0.38 (0.32, 0.46) | <0.001 |
| Graduate and above | 0.16 (0.07, 0.36) | <0.001 |
| BMI (kg/m^2^) | 0.91 (0.89, 0.93) | <0.001 |
| Current smokers, n(%) |  |  |
| No | *Ref* |  |
| Yes | 1.42 (1.20, 1.67) | <0.001 |
| Current drinkers, n(%) |  |  |
| No | *Ref* |  |
| Yes | 0.87 (0.73, 1.03) | 0.111 |
| Physical activity levels, n(%) |  |  |
| Low | *Ref* | 0.947 |
| Middle | 0.73 (0.60, 0.89) | 0.002 |
| High | 0.47 (0.39, 0.57) | <0.001 |
| Sleep duration on workdays (hours) | 0.93 (0.89, 0.98) | 0.008 |
| Sleep duration on non-workdays (hours) | 0.94 (0.89, 0.99) | 0.028 |
| SBP (mmHg) | 1.02 (1.01, 1.02) | <0.001 |
| DBP (mmHg) | 1.01 (1.01, 1.02) | <0.001 |
| History of myocardial infarction, n(%) |  |  |
| No | *Ref* |  |
| Yes | 1.06 (0.46, 2.44) | 0.887 |
| History of stroke, n(%) |  |  |
| No | *Ref* |  |
| Yes | 1.81 (1.18, 2.78) | 0.007 |
| ACEIs or ARBs, n(%) |  |  |
| No | *Ref* |  |
| Yes | 1.13 (0.72, 1.75) | 0.598 |
| Beta blockers, n(%) |  |  |
| No | *Ref* |  |
| Yes | 0.75 (0.23, 2.39) | 0.625 |
| Calcium channel blockers, n(%) |  |  |
| No | *Ref* |  |
| Yes | 1.03 (0.77, 1.38) | 0.836 |
| Diuretics, n(%) |  |  |
| No | *Ref* |  |
| Yes | 3.00 (0.86, 10.47) | 0.085 |
| Abbreviations: SBP, systolic blood pressure; DBP, diastolic blood pressure; ACEIs, angiotensin-converting enzyme inhibitors; ARBs, angiotensin receptor blockers; *Ref*, reference; *HR*, hazard ratio; *CI*, confidence interval. | | |

| **Supplementary Table 2.** Characteristics of the study population grouped by age or sex. | | | | | | | |  |
| --- | --- | --- | --- | --- | --- | --- | --- | --- |
| Characteristics | Age (years) | | *P-value* |  | Sex | | *P-value* | |
|  | <60 | ≥60 |  |  | Male | Female |  |  |
| Number of subjects (n) | 6169 | 5948 |  |  | 4857 | 7260 |  | |
| Age (years) | 48.12 ± 6.77 | 70.37 ± 7.49 | <0.001 |  | 59.99 ± 13.39 | 58.41 ± 13.06 | <0.001 | |
| Male, n(%) | 2256 (36.57%) | 2601 (43.73%) | <0.001 |  |  |  |  | |
| SBP (mmHg) | 122.41 ± 16.35 | 133.72 ± 21.04 | <0.001 |  | 129.21 ± 18.45 | 127.10 ± 20.33 | <0.001 | |
| DBP (mmHg) | 74.76 ± 10.60 | 74.84 ± 10.89 | 0.254 |  | 76.94 ± 10.62 | 73.36 ± 10.59 | <0.001 | |
| BMR(kcal/day) | 1280.86 ± 188.65 | 1221.64 ± 186.71 | <0.001 |  | 1376.45 ± 187.31 | 1168.39 ± 139.42 | <0.001 | |
| BMI (kg/m^2^) | 23.43 ± 3.56 | 22.69 ± 3.70 | <0.001 |  | 22.90 ± 3.71 | 23.18 ± 3.60 | <0.001 | |
| BMI group (kg/m^^2^) |  |  | <0.001 |  |  |  | <0.001 | |
| Underweight (<18.5) | 276 (4.48%) | 628 (10.57%) |  |  | 413 (8.50%) | 491 (6.77%) |  | |
| Normal weight (≥18.5, <24) | 3392 (55.01%) | 3395 (57.13%) |  |  | 2745 (56.53%) | 4042 (55.73%) |  | |
| Overweight (≥24, <28) | 1962 (31.82%) | 1477 (24.85%) |  |  | 1351 (27.82%) | 2088 (28.79%) |  | |
| General obesity (≥28) | 536 (8.69%) | 443 (7.45%) |  |  | 347 (7.15%) | 632 (8.71%) |  | |
| Waist circumference (cm) | 80.02 ± 8.97 | 79.77 ± 9.32 | 0.089 |  | 81.11 ± 9.07 | 79.09 ± 9.10 | <0.001 | |
| Urban residence, n(%) | 2728 (44.22%) | 3471 (58.36%) | <0.001 |  | 2521 (51.90%) | 3678 (50.66%) | 0.18 | |
| Education level, n(%) |  |  | <0.001 |  |  |  | <0.001 | |
| Primary school or below | 2740 (44.82%) | 4262 (72.80%) |  |  | 2274 (47.22%) | 4728 (66.12%) |  | |
| Middle school | 3086 (50.48%) | 1491 (25.47%) |  |  | 2303 (47.82%) | 2274 (31.80%) |  | |
| Graduate and above | 287 (4.69%) | 101 (1.73%) |  |  | 239 (4.96%) | 149 (2.08%) |  | |
| Current smokers, n(%) | 1191 (19.37%) | 1147 (19.31%) | 0.934 |  | 2186 (45.18%) | 152 (2.10%) | <0.001 | |
| Current drinkers, n(%) | 1675 (27.27%) | 1340 (22.58%) | <0.001 |  | 2009 (41.51%) | 1006 (13.90%) | <0.001 | |
| Physical activity levels, n(%) |  |  | <0.001 |  |  |  | <0.001 | |
| Low | 802 (13.00%) | 1030 (17.32%) |  |  | 992 (20.50%) | 840 (11.59%) |  | |
| Middle | 1523 (24.69%) | 1685 (28.33%) |  |  | 1254 (25.91%) | 1954 (26.96%) |  | |
| High | 3822 (61.95%) | 3225 (54.22%) |  |  | 2594 (53.60%) | 4453 (61.45%) |  | |
| Sleep duration on workdays (hours) | 7.39 ± 1.22 | 7.19 ± 1.40 | <0.001 |  | 7.29 ± 1.30 | 7.29 ± 1.32 | 0.544 | |
| Sleep duration on non-workdays (hours) | 7.71 ± 1.27 | 7.40 ± 1.43 | <0.001 |  | 7.56 ± 1.33 | 7.56 ± 1.38 | 0.573 | |
| Hypertension, n(%) | 1217 (19.73%) | 2920 (49.09%) | <0.001 |  | 1707 (35.15%) | 2430 (33.47%) | 0.057 | |
| History of myocardial infarction, n(%) | 15 (0.24%) | 70 (1.18%) | <0.001 |  | 40 (0.82%) | 45 (0.62%) | 0.188 | |
| History of stroke, n(%) | 34 (0.55%) | 178 (2.99%) | <0.001 |  | 107 (2.20%) | 105 (1.45%) | 0.002 | |
| ACEIs or ARBs, n(%) | 108 (1.75%) | 188 (3.16%) | <0.001 |  | 129 (2.66%) | 167 (2.30%) | 0.214 | |
| Beta blockers, n(%) | 16 (0.26%) | 43 (0.72%) | <0.001 |  | 28 (0.58%) | 31 (0.43%) | 0.247 | |
| Calcium channel blockers, n(%) | 237 (3.84%) | 536 (9.01%) | <0.001 |  | 322 (6.63%) | 451 (6.21%) | 0.357 | |
| Diuretics, n(%) | 6 (0.10%) | 11 (0.18%) | 0.197 |  | 10 (0.21%) | 7 (0.10%) | 0.115 | |
| Other agents, n(%) | 36 (0.58%) | 84 (1.41%) | <0.001 |  | 49 (1.01%) | 71 (0.98%) | 0.866 | |
|  |  |  |  |  |  |  |  | |
| Follow-up |  |  |  |  |  |  |  | |
| Median follow-up time, years | 5.59 (5.31-5.68) | 5.61 (5.27-5.74) | 0.012 |  | 5.58 (5.27-5.72) | 5.61 (5.31-5.75) | <0.001 | |
| All-cause mortality, n(%) | 126 (2.04%) | 683 (11.48%) | <0.001 |  | 478 (9.84%) | 331 (4.56%) | <0.001 | |
| Cause of death, n(%) |  |  | 0.006 |  |  |  | <0.001 | |
| Stroke | 14 (11.11%) | 116 (16.98%) |  |  | 80 (1.65%) | 50 (0.69%) |  | |
| Cardiovascular disease | 33 (26.19%) | 209 (30.60%) |  |  | 134 (2.76%) | 108 (1.49%) |  | |
| Malignant tumor | 21 (16.67%) | 51 (7.47%) |  |  | 42 (0.86%) | 30 (0.41%) |  | |
| Respiratory failure | 13 (10.32%) | 98 (14.35%) |  |  | 75 (1.54%) | 36 (0.50%) |  | |
| Others | 27 (21.43%) | 107 (15.67%) |  |  | 83 (1.71%) | 51 (0.70%) |  | |
| Unknown | 18 (14.29%) | 102 (14.93%) |  |  | 64 (1.32%) | 56 (0.77%) |  | |
| Abbreviations: BMR, basal metabolic rate; BMI, body mass index; SBP, systolic blood pressure; DBP, diastolic blood pressure; ACEIs, angiotensin-converting enzyme inhibitors; ARBs, angiotensin receptor blockers. | | | | | | | |  |

| **Supplementary Table 3.** Threshold effect analysis of age and basal metabolic rate using Piece-wise logistic regression. | | | | | | |
| --- | --- | --- | --- | --- | --- | --- |
| **Inflection point of age** | **No. of participants** |  | **Effect size (β)** | **95% CI** | **P-value** | **LLR test** |
| <61years | 6475 |  | -0.71 | (-1.20, -0.22) | 0.005 | <0.001 |
| ≥61years | 5642 |  | -5.21 | (-5.77, -4.65) | <0.001 |  |
| Effect: Basal metabolic rate; Cause: age.  Abbreviations: CI, confidence interval; LLR, log-likelihood ratio. | | | | | | |
|  |  |  |  |  |  |  |

| **Supplementary table 4.** Hazard ratios of different BMR categories for all-cause mortality excluded of subjects with history of myocardial infarction and stroke. | | | | | | | | | | | | | | | | |  |
| --- | --- | --- | --- | --- | --- | --- | --- | --- | --- | --- | --- | --- | --- | --- | --- | --- | --- |
| Variables | Event, n(%) | Crude Model | | |  | Model Ⅰ | | |  | Model Ⅱ | | | | | | | |
|  |  | *HR (95%CI)* | *P-value* | |  | *HR (95%CI)* | *P-value* | |  | Multivariable analysis ⃰ | | |  | Propensity-score analysis ʃ | | | |
|  |  |  |  |  |  |  |  |  |  | *HR (95%CI)* | *P-value* | |  | *HR (95%CI)* | *P-value* | | |
| Male |  |  |  | |  |  |  | |  |  |  | |  |  |  | | |
| Age <60years |  |  |  | |  |  |  | |  |  |  | |  |  |  | | |
| BMR (kcal/day) |  |  |  | |  |  |  | |  |  |  | |  |  |  | | |
| Per SD increase | 70 (3.13%) | 0.83 (0.66, 1.04) | 0.111 | |  | 0.83 (0.65, 1.05) | 0.120 | |  | 0.92 (0.70, 1.21) | 0.566 | |  | 0.95 (0.73, 1.25) | 0.701 | | |
| Quartiles of BMR |  |  |  | |  |  |  | |  |  |  | |  |  |  | | |
| Q1 [843, 1112] | 3 (2.11%) | *Ref* |  | |  | *Ref* |  | |  | *Ref* |  | |  | *Ref* |  | | |
| Q2 [1120, 1217] | 12 (5.29%) | 2.40 (0.68, 8.49) | 0.176 | |  | 2.36 (0.67, 8.36) | 0.184 | |  | 2.21 (0.61, 7.97) | 0.226 | |  | 2.33 (0.71, 8.03) | 0.294 | | |
| Q3 [1218, 1367] | 20 (4.77%) | 2.56 (0.76, 8.61) | 0.130 | |  | 2.21 (0.66, 7.48) | 0.201 | |  | 2.26 (0.66, 7.68) | 0.192 | |  | 2.35 (0.78, 7.79) | 0.212 | | |
| Q4 [1368, 1789] | 35 (2.42%) | 1.34 (0.41, 4.35) | 0.630 | |  | 1.23 (0.38, 4.03) | 0.726 | |  | 1.41 (0.43, 4.64) | 0.575 | |  | 1.54 (0.55, 4.78) | 0.621 | | |
| P for trend |  | 0.171 | | |  | 0.129 | | |  | 0.458 | | |  | 0.612 | | | |
| Age ≥60years |  |  |  | |  |  | |  |  |  | |  |  |  | |  | |
| BMR (kcal/day) |  |  |  | |  |  | |  |  |  | |  |  |  | |  | |
| Per SD increase | 386 (15.56%) | 0.62 (0.55, 0.69) | <0.001 | |  | 0.74 (0.66, 0.83) | | <0.001 |  | 0.81 (0.71, 0.93) | | 0.002 |  | 0.86 (0.77, 0.98) | | 0.003 | |
| Quartiles of BMR |  |  |  | |  |  | |  |  |  | |  |  |  | |  | |
| Q1 [850, 1115] | 73 (27.97%) | *Ref* |  | |  | *Ref* | |  |  | *Ref* | |  |  | *Ref* | |  | |
| Q2 [1116, 1217] | 95 (20.52%) | 0.68 (0.50, 0.93) | 0.014 | |  | 0.79 (0.58, 1.07) | | 0.130 |  | 0.78 (0.57, 1.06) | | 0.117 |  | 0.81 (0.59, 1.11) | | 0.131 | |
| Q3 [1218, 1367] | 119 (15.39%) | 0.50 (0.38, 0.67) | <0.001 | |  | 0.65 (0.48, 0.87) | | 0.004 |  | 0.68 (0.50, 0.92) | | 0.012 |  | 0.72 (0.57, 0.98) | | 0.021 | |
| Q4 [1368, 1788] | 99 (10.06%) | 0.31 (0.23, 0.42) | <0.001 | |  | 0.49 (0.36, 0.67) | | <0.001 |  | 0.62 (0.44, 0.87) | | 0.006 |  | 0.66 (0.47, 0.91) | | 0.009 | |
| P for trend |  | <0.001 | | |  | <0.001 | | |  | 0.008 | | |  | 0.023 | | | |
|  |  |  | |  |  |  | |  |  |  | |  |  |  | |  | |
| Female |  |  | |  |  |  | |  |  |  | |  |  |  | |  | |
| Age <60years |  |  | |  |  |  | |  |  |  | |  |  |  | |  | |
| BMR (kcal/day) |  |  | |  |  |  | |  |  |  | |  |  |  | |  | |
| Per SD increase | 54 (1.39%) | 0.85 (0.56, 1.29) | | 0.448 |  | 0.85 (0.56, 1.29) | | 0.438 |  | 0.95 (0.57, 1.57) | | 0.828 |  | 0.97 (0.59, 1.58) | | 0.837 | |
| Quartiles of BMR |  |  | |  |  |  | |  |  |  | |  |  |  | |  | |
| Q1 [797, 1115] | 15 (1.42%) | *Ref* | |  |  | *Ref* | |  |  | *Ref* | |  |  | *Ref* | |  | |
| Q2 [1116, 1217] | 20 (1.50%) | 1.02 (0.52, 2.00) | | 0.945 |  | 1.02 (0.52, 1.99) | | 0.959 |  | 1.19 (0.59, 2.39) | | 0.634 |  | 1.25 (0.65, 2.82) | | 0.703 | |
| Q3 [1218, 1367] | 16 (1.40%) | 0.97 (0.48, 1.96) | | 0.934 |  | 0.96 (0.47, 1.94) | | 0.912 |  | 1.14 (0.50, 2.58) | | 0.754 |  | 1.18 (0.54, 2.89) | | 0.812 | |
| Q4 [1368, 1771] | 3 (0.86%) | 0.59 (0.17, 2.03) | | 0.401 |  | 0.57 (0.17, 1.98) | | 0.378 |  | 0.73 (0.18, 2.95) | | 0.657 |  | 0.77 (0.22, 3.20) | | 0.712 | |
| P for trend |  | 0.475 | | |  | 0.446 | | |  | 0.813 | | |  | 0.924 | | | |
| Age ≥60years |  |  | |  |  |  | |  |  |  | |  |  |  | |  | |
| BMR (kcal/day) |  |  | |  |  |  | |  |  |  | |  |  |  | |  | |
| Per SD increase | 271 (8.40%) | 0.74 (0.63, 0.87) | | <0.001 |  | 0.96 (0.83, 1.13) | | 0.647 |  | 1.03 (0.87, 1.22) | | 0.741 |  | 1.09 (0.92, 1.57) | | 0.812 | |
| Quartiles of BMR |  |  | |  |  |  | |  |  |  | |  |  |  | |  | |
| Q1 [787, 1115] | 150 (10.13%) | *Ref* | |  |  | *Ref* | |  |  | *Ref* | |  |  | *Ref* | |  | |
| Q2 [1116, 1217] | 64 (6.91%) | 0.67 (0.50, 0.90) | | 0.008 |  | 0.91 (0.67, 1.22) | | 0.527 |  | 0.94 (0.69, 1.30) | | 0.723 |  | 0.98 (0.73, 1.62) | | 0.758 | |
| Q3 [1218, 1367] | 47 (7.31%) | 0.71 (0.51, 0.99) | | 0.044 |  | 1.04 (0.74, 1.46) | | 0.816 |  | 1.19 (0.82, 1.72) | | 0.352 |  | 1.24 (0.87, 1.77) | | 0.342 | |
| Q4 [1368, 1761] | 10 (5.65%) | 0.54 (0.29, 1.03) | | 0.061 |  | 0.71 (0.37, 1.34) | | 0.287 |  | 0.81 (0.42, 1.57) | | 0.528 |  | 0.87 (0.48, 1.63) | | 0.613 | |
| P for trend |  | 0.005 | | |  | 0.485 | | |  | 0.983 | | |  | 0.992 | | | |
| Abbreviations: BMR, basal metabolic rate; *Ref*, reference; HR, hazard ratio; CI, confidence interval; SD, standard deviation. Model Ⅰ adjusted for age.  Model Ⅱ: ⃰ Shown are the hazard ratios from the multivariable Cox proportional-hazards model with additional adjustment for age, SBP, DBP, BMI, current smokers, sleep duration on workdays or non-workdays. ʃ Shown are the hazard ratios from the multivariable Cox proportional-hazards model with the same covariates with inverse probability weighting according to the propensity score. | | | | | | | | | | | | | | | | | |

| **Supplementary table 5. Hazard ratios of different BMR categories for all-cause mortality excluded of subjects less than one year of follow up.** | | | | | | | | | | | | | | | | |  |
| --- | --- | --- | --- | --- | --- | --- | --- | --- | --- | --- | --- | --- | --- | --- | --- | --- | --- |
| Variables | Event, n(%) | Crude Model | | | |  | Model Ⅰ | |  | Model Ⅱ | | | | | | | |
|  |  | *HR (95%CI)* | | *P-value* | |  | *HR (95%CI)* | *P-value* |  | Multivariable analysis ⃰ | | |  | Propensity-score analysis ʃ | | | |
|  |  |  |  |  |  |  |  |  |  | *HR (95%CI)* | | *P-value* |  | *HR (95%CI)* | *P-value* | | |
| Male |  |  | |  | |  |  |  |  |  | |  |  |  |  | | |
| Age <60years |  |  | |  | |  |  |  |  |  | |  |  |  |  | | |
| BMR (kcal/day) |  |  | |  | |  |  |  |  |  | |  |  |  |  | | |
| Per SD increase | 67 (2.98%) | 0.86 (0.67, 1.09) | | 0.198 | |  | 0.86 (0.67, 1.09) | 0.210 |  | 0.91 (0.69, 1.19) | | 0.497 |  | 0.93 (0.71, 1.21) | 0.583 | | |
| Quartiles of BMR | |  | |  | |  |  |  |  |  | |  |  |  |  | | |
| Q1 [843, 1112] | 3 (2.13%) | *Ref* | |  | |  | *Ref* |  |  | *Ref* | |  |  | *Ref* |  | | |
| Q2 [1120, 1219] | 10 (4.41%) | 1.97 (0.54, 7.18) | | 0.302 | |  | 1.93 (0.53, 7.01) | 0.318 |  | 1.86 (0.50, 6.91) | | 0.354 |  | 1.89 (0.53, 6.95) | 0.418 | | |
| Q3 [1220, 1368] | 20 (4.75%) | 2.54 (0.76, 8.57) | | 0.132 | |  | 2.18 (0.65, 7.38) | 0.209 |  | 2.24 (0.66, 7.61) | | 0.197 |  | 2.28 (0.70, 7.65) | 0.216 | | |
| Q4 [1369, 1789] | 34 (2.34%) | 1.30 (0.40, 4.23) | | 0.668 | |  | 1.19 (0.36, 3.87) | 0.778 |  | 1.25 (0.38, 4.11) | | 0.713 |  | 1.29 (0.46, 4.19) | 0.826 | | |
| P for trend |  | 0.261 | | | |  | 0.198 | |  | 0.369 | | |  | 0.401 | | | |
| Age ≥60years |  |  | |  | |  |  |  |  |  | |  |  |  | |  | |
| BMR (kcal/day) |  |  | |  | |  |  |  |  |  | |  |  |  | |  | |
| Per SD increase | 367 (14.41%) | 0.65 (0.57, 0.72) | | <0.001 | | | 0.77 (0.68, 0.86) | <0.001 |  | 0.82 (0.72, 0.94) | | 0.003 |  | 0.85 (0.76, 0.103) | | 0.004 | |
| Quartiles of BMR | |  | |  | |  |  |  |  |  | |  |  |  | |  | |
| Q1 [848, 1115] | 63 (24.32%) | *Ref* | |  | |  | *Ref* |  |  | *Ref* | |  |  | *Ref* | |  | |
| Q2 [1116, 1218] | 86 (18.49%) | 0.72 (0.52, 0.99) | | 0.043 | |  | 0.82 (0.59, 1.13) | 0.230 |  | 0.81 (0.58, 1.12) | | 0.206 |  | 0.85 (0.62, 1.16) | | 0.312 | |
| Q3 [1220, 1368] | 118 (14.84%) | 0.57 (0.42, 0.78) | | <0.001 | | | 0.73 (0.53, 0.99) | 0.043 |  | 0.74 (0.54, 1.02) | | 0.062 |  | 0.78 (0.58, 1.06) | | 0.071 | |
| Q4 [1369, 1788] | 100 (9.74%) | 0.36 (0.26, 0.49) | | <0.001 | | | 0.55 (0.40, 0.76) | <0.001 |  | 0.66 (0.46, 0.93) | | 0.018 |  | 0.69 (0.49, 0.96) | | 0.022 | |
| P for trend |  | <0.001 | | |  | | <0.001 | |  | 0.024 | | |  | 0.035 | | | |
|  |  |  | |  |  | |  |  |  |  |  | |  |  |  | | |
| Female |  |  | |  |  | |  |  |  |  |  | |  |  |  | | |
| Age <60years |  |  | |  |  | |  |  |  |  |  | |  |  |  | | |
| BMR (kcal/day) |  |  | |  |  | |  |  |  |  |  | |  |  |  | | |
| Per SD increase | 49 (1.26%) | 0.89 (0.57, 1.37) | | 0.583 |  | | 0.88 (0.57, 1.36) | 0.571 |  | 0.83 (0.48, 1.42) | 0.494 | |  | 0.86 (0.51, 1.45) | 0.512 | | |
| Quartiles of BMR | |  | |  |  | |  |  |  |  |  | |  |  |  | | |
| Q1 [797, 1115] | 12 (1.14%) | *Ref* | |  |  | | *Ref* |  |  | *Ref* |  | |  | *Ref* |  | | |
| Q2 [1116, 1219] | 19 (1.40%) | 1.19 (0.58, 2.44) | | 0.643 |  | | 1.18 (0.57, 2.42) | 0.660 |  | 1.18 (0.56, 2.50) | 0.668 | |  | 1.21 (0.59, 2.53) | 0.703 | | |
| Q3 [1220, 1368] | 16 (1.41%) | 1.22 (0.58, 2.58) | | 0.600 |  | | 1.21 (0.57, 2.55) | 0.622 |  | 1.15 (0.48, 2.73) | 0.756 | |  | 1.19 (0.51, 2.77) | 0.821 | | |
| Q4 [1369, 1771] | 2 (0.58%) | 0.49 (0.11, 2.21) | | 0.356 |  | | 0.48 (0.11, 2.15) | 0.338 |  | 0.42 (0.08, 2.23) | 0.310 | |  | 0.45 (0.11, 2.25) | 0.323 | | |
| P for trend |  | 0.585 | | |  | | 0.585 | |  | 0.496 | | |  | 0.523 | | | |
| Age ≥60years |  |  |  | |  | |  |  |  |  |  | |  |  | |  | |
| BMR (kcal/day) |  |  |  | |  | |  |  |  |  |  | |  |  | |  | |
| Per SD increase | 258 (7.80%) | 0.74 (0.62, 0.87) | <0.001 | | | | 0.97 (0.83, 1.13) | 0.686 |  | 1.01 (0.84, 1.21) | 0.932 | |  | 1.03 (0.87, 1.24) | | 0.956 | |
| Quartiles of BMR | |  |  | |  | |  |  |  |  |  | |  |  | |  | |
| Q1 [787, 1115] | 144 (9.46%) | *Ref* |  | |  | | *Ref* |  |  | *Ref* |  | |  | *Ref* | |  | |
| Q2 [1116, 1219] | 60 (6.31%) | 0.65 (0.48, 0.89) | 0.006 | |  | | 0.88 (0.65, 1.20) | 0.433 |  | 0.91 (0.66, 1.26) | 0.574 | |  | 0.94 (0.69, 1.28) | | 0.602 | |
| Q3 [1220, 1368] | 44 (6.67%) | 0.70 (0.50, 0.98) | 0.036 | |  | | 1.01 (0.72, 1.43) | 0.940 |  | 1.10 (0.75, 1.62) | 0.620 | |  | 1.14 (0.79, 1.66) | | 0.634 | |
| Q4 [1369, 1761] | 10 (5.68%) | 0.58 (0.31, 1.10) | 0.096 | |  | | 0.76 (0.40, 1.45) | 0.407 |  | 0.84 (0.43, 1.63) | 0.603 | |  | 0.88 (0.47, 1.67) | | 0.712 | |
| P for trend |  | 0.523 | | |  | | 0.523 | |  | 0.879 | | |  | 0.934 | | | |
| Abbreviations: BMR, basal metabolic rate; *Ref*, reference; HR, hazard ratio; CI, confidence interval; SD, standard deviation. Model Ⅰ adjusted for age.  Model Ⅱ: ⃰ Shown are the hazard ratios from the multivariable Cox proportional-hazards model with additional adjustment for age, SBP, DBP, BMI, education level, current smokers, sleep duration on workdays or non-workdays. ʃ Shown are the hazard ratios from the multivariable Cox proportional-hazards model with the same covariates with inverse probability weighting according to the propensity score. | | | | | | | | | | | | | | | | | |

| **Supplementary table 6. Hazard ratios of different BMR categories for all-cause mortality grouped by BMI levels.** | | | | | | | | | | | | | | | | | | | |
| --- | --- | --- | --- | --- | --- | --- | --- | --- | --- | --- | --- | --- | --- | --- | --- | --- | --- | --- | --- |
| Variables | | Event, n(%) | | Crude Model | | |  | | Model Ⅰ | | | |  | Model Ⅱ | | | | |  |
|  |  |  |  | *HR (95%CI)* | *P-value* | |  |  | *HR (95%CI)* | | *P-value* | |  | *HR (95%CI)* | | | *P-value* | |  |
| Underweight | |  | |  |  | |  | |  | |  | |  |  | | |  | |  |
| BMR (kcal/day) | |  | |  |  | |  | |  | |  | |  |  | | |  | |  |
| Per SD increase | | 124 (13.72%) | | 1.02 (0.85, 1.22) | 0.851 | |  | | 0.96 (0.77, 1.19) | | 0.697 | |  | 0.97 (0.77, 1.20) | | | 0.753^a^ | |  |
| Quartiles of BMR | |  | |  |  | |  | |  | |  | |  |  | | |  | |  |
| Q1 [790, 1115] | | 62 (12.86%) | | *Ref* |  | |  | | *Ref* | |  | |  | *Ref* | | |  | |  |
| Q2 [1116, 1218] | | 33 (16.92%) | | 1.36 (0.89, 2.07) | 0.158 | |  | | 0.84 (0.52, 1.34) | | 0.463 | |  | 0.84 (0.52, 1.36) | | | 0.470^a^ | |  |
| Q3 [1220, 1367] | | 18 (10.98%) | | 0.82 (0.48, 1.38) | 0.448 | |  | | 0.76 (0.43, 1.34) | | 0.346 | |  | 0.79 (0.44, 1.42) | | | 0.436^a^ | |  |
| Q4 [1368, 1773] | | 11 (17.46%) | | 1.30 (0.68, 2.46) | 0.429 | |  | | 1.08 (0.55, 2.12) | | 0.823 | |  | 1.04 (0.52, 2.07) | | | 0.908^a^ | |  |
| P for trend | |  | | 0.760 | | |  | | 0.851 | | | |  | 0.863 | | | | |  |
| Normal weight | |  | |  |  | |  | |  | |  | |  |  | | |  | |  |
| BMR (kcal/day) | |  | |  |  | |  | |  | |  | |  |  | | |  | |  |
| Per SD increase | | 487 (7.18%) | | 0.99 (0.89, 1.10) | 0.862 | |  | | 0.89 (0.78, 1.02) | | 0.089 | |  | 0.92 (0.81, 1.05) | | | 0.215^b^ | |  |
| Quartiles of BMR | |  | |  |  | |  | |  | |  | |  |  | | |  | |  |
| Q1 [795, 1115] | | 143 (6.63%) | | *Ref* |  | |  | | *Ref* | |  | |  | *Ref* | | |  | |  |
| Q2 [1116, 1219] | | 126 (6.38%) | | 0.95 (0.75, 1.21) | 0.668 | |  | | 1.09 (0.85, 1.41) | | 0.504 | |  | 1.09 (0.84, 1.41) | | | 0.529^b^ | |  |
| Q3 [1220, 1367] | | 146 (10.08%) | | 1.59 (1.27, 2.01) | <0.001 | |  | | 1.07 (0.81, 1.41) | | 0.651 | |  | 1.09 (0.82, 1.44) | | | 0.563^b^ | |  |
| Q4 [1368, 1774] | | 72 (5.97%) | | 0.91 (0.69, 1.21) | 0.518 | |  | | 0.78 (0.55, 1.09) | | 0.139 | |  | 0.85 (0.61, 1.19) | | | 0.352^b^ | |  |
| P for trend | |  | | 0.476 | | |  | | 0.105 | | | |  | 0.325 | | | | |  |
| Overweight | |  | |  |  | |  | |  | |  | |  |  | | |  | |  |
| BMR (kcal/day) | |  | |  |  | |  | |  | |  | |  |  | | |  | |  |
| Per SD increase | | 163 (4.74%) | | 0.87 (0.74, 1.03) | 0.098 | |  | | 0.77 (0.64, 0.92) | | 0.005 | |  | 0.80 (0.66, 0.98) | | | 0.027^b^ | |  |
| Quartiles of BMR | |  | |  |  | |  | |  | |  | |  |  | | |  | |  |
| Q1 [801, 1115] | | 32 (10.03%) | | *Ref* |  | |  | | *Ref* | |  | |  | *Ref* | | |  | |  |
| Q2 [1116, 1219] | | 31 (3.94%) | | 0.39 (0.24, 0.64) | <0.001 | |  | | 0.62 (0.37, 1.02) | | 0.059 | |  | 0.61 (0.36, 1.03) | | | 0.067^b^ | |  |
| Q3 [1220, 1367] | | 40 (3.62%) | | 0.35 (0.22, 0.56) | <0.001 | |  | | 0.65 (0.40, 1.04) | | 0.072 | |  | 0.71 (0.44, 1.16) | | | 0.175^b^ | |  |
| Q4 [1368, 1787] | | 60 (4.88%) | | 0.49 (0.32, 0.76) | 0.001 | |  | | 0.43 (0.26, 0.72) | | 0.001 | |  | 0.47 (0.28, 0.79) | | | 0.004^b^ | |  |
| P for trend | |  | | 0.164 | | |  | | 0.003 | | | |  | 0.012 | | | | |  |
| General obesity | |  | |  |  | |  | |  | |  | |  |  | | |  | |  |
| BMR (kcal/day) | |  | |  |  | |  | |  | |  | |  |  | | |  | |  |
| Per SD increase | | 35 (3.58%) | | 0.55 (0.40, 0.76) | <0.001 | |  | | 0.63 (0.46, 0.86) | | 0.004 | |  | 0.68 (0.48, 0.97) | | | 0.033^b^ | |  |
| Quartiles of BMR | |  | |  |  | |  | |  | |  | |  |  | | |  | |  |
| Q1 [787, 1112] | | 8 (16.00%) | | *Ref* |  | |  | | *Ref* | |  | |  | *Ref* | | |  | |  |
| Q2 [1116, 1219] | | 7 (8.14%) | | 0.44 (0.16, 1.23) | 0.117 | |  | | 0.71 (0.25, 1.99) | | 0.514 | |  | 0.71 (0.23, 2.20) | | | 0.558^b^ | |  |
| Q3 [1220, 1367] | | 9 (3.02%) | | 0.17 (0.07, 0.45) | <0.001 | |  | | 0.32 (0.11, 0.91) | | 0.032 | |  | 0.40 (0.13, 1.22) | | | 0.107^b^ | |  |
| Q4 [1368, 1789] | | 11 (2.02%) | | 0.13 (0.05, 0.32) | <0.001 | |  | | 0.23 (0.09, 0.61) | | 0.003 | |  | 0.26 (0.09, 0.75) | | | 0.013^b^ | |  |
| P for trend | |  | | <0.001 | | |  | | 0.002 | | | |  | 0.010 | | | | |  |
| Abbreviations: BMI, body mass index; BMR, basal metabolic rate; *Ref*, reference; *HR*, hazard ratio; *CI*, confidence interval; SD, standard deviation. Model Ⅰ adjusted for age and gender.  Model Ⅱ ^a^ adjusted for age, gender, SBP, DBP, education level, current smokers, physical activity levels, sleep duration on non-workdays, history of myocardial infarction and Beta blockers. ^b^ adjusted for age, gender, SBP, DBP, BMI, education level, current smokers and drinkers, physical activity levels, sleep duration on workdays or non-workdays, history of stroke, diuretics and calcium channel blockers usage. | | | | | | | | | | | | | | | | | | |  |
| **Supplementary table 7.** Hazard ratios of different BMR categories for all-cause mortality grouped by age strata. | | | | | | | | | | | | | | | | | | | |
| Variables | Event, n(%) | | Crude Model | | | | |  | | Model Ⅰ | | | | |  | Model Ⅱ | | | |
|  |  |  | *HR (95%CI)* | | | *P-value* | |  |  | *HR (95%CI)* | | *P-value* | | |  | *HR (95%CI)* | | *P-value* | |
| Age [35, 44] |  | |  | | |  | |  | |  | |  | | |  |  | |  | |
| BMR (kcal/day) |  | |  | | |  | |  | |  | |  | | |  |  | |  | |
| Per SD increase | 24 (1.20%) | | 0.91 (0.60, 1.36) | | | 0.637 | |  | | 0.66 (0.42, 1.06) | | 0.086 | | |  | 0.74 (0.46, 1.20) | | 0.222 | |
| Quartiles of BMR |  | |  | | |  | |  | |  | |  | | |  |  | |  | |
| Q1 [871, 1115] | 3 (0.70%) | | *Ref* | | |  | |  | | *Ref* | |  | | |  | *Ref* | |  | |
| Q2 [1116, 1219] | 10 (1.95%) | | 2.57 (0.71, 9.37) | | | 0.151 | |  | | 2.39 (0.65, 8.72) | | 0.187 | | |  | 2.67 (0.73, 9.84) | | 0.139 | |
| Q3 [1220, 1367] | 4 (0.85%) | | 1.19 (0.27, 5.34) | | | 0.817 | |  | | 1.00 (0.22, 4.53) | | 0.999 | | |  | 1.13 (0.25, 5.20) | | 0.872 | |
| Q4 [1368, 1789] | 7 (1.20%) | | 1.67 (0.43, 6.46) | | | 0.458 | |  | | 0.84 (0.19, 3.72) | | 0.820 | | |  | 1.14 (0.25, 5.12) | | 0.867 | |
| P for trend |  | | 0.946 | | | | |  | | 0.297 | | | | |  | 0.587 | | | |
| Age [45, 54] |  | |  | | |  | |  | |  | |  | | |  |  | |  | |
| BMR (kcal/day) |  | |  | | |  | |  | |  | |  | | |  |  | |  | |
| Per SD increase | 55 (1.96%) | | 1.14 (0.87, 1.48) | | | 0.350 | |  | | 0.82 (0.60, 1.11) | | 0.192 | | |  | 0.86 (0.63, 1.17) | | 0.329 | |
| Quartiles of BMR |  | |  | | |  | |  | |  | |  | | |  |  | |  | |
| Q1 [797, 1115] | 8 (1.58%) | | *Ref* | | |  | |  | | *Ref* | |  | | |  | *Ref* | |  | |
| Q2 [1116, 1219] | 13 (1.74%) | | 1.12 (0.46, 2.69) | | | 0.806 | |  | | 1.13 (0.47, 2.73) | | 0.782 | | |  | 1.15 (0.48, 2.78) | | 0.758 | |
| Q3 [1220, 1367] | 18 (2.51%) | | 1.62 (0.70, 3.72) | | | 0.259 | |  | | 1.37 (0.59, 3.16) | | 0.467 | | |  | 1.39 (0.60, 3.23) | | 0.445 | |
| Q4 [1368, 1785] | 16 (1.92%) | | 1.33 (0.57, 3.12) | | | 0.508 | |  | | 0.59 (0.24, 1.48) | | 0.266 | | |  | 0.66 (0.26, 1.67) | | 0.383 | |
| P for trend |  | | 0.454 | | | | |  | | 0.152 | | | | |  | 0.246 | | | |
| Age [55, 64] |  | |  | | |  | |  | |  | |  | | |  |  | |  | |
| BMR (kcal/day) |  | |  | | |  | |  | |  | |  | | |  |  | |  | |
| Per SD increase | 110 (3.71%) | | 1.16 (0.96, 1.40) | | | 0.130 | |  | | 0.80 (0.64, 1.00) | | 0.055 | | |  | 0.95 (0.75, 1.21) | | 0.700 | |
| Quartiles of BMR |  | |  | | |  | |  | |  | |  | | |  |  | |  | |
| Q1 [832 1115] | 21 (3.33%) | | *Ref* | | |  | |  | | *Ref* | |  | | |  | *Ref* | |  | |
| Q2 [1116, 1219] | 19 (2.58%) | | 0.74 (0.40, 1.38) | | | 0.342 | |  | | 0.64 (0.34, 1.19) | | 0.157 | | |  | 0.66 (0.34, 1.27) | | 0.210 | |
| Q3 [1220, 1367] | 34 (4.30%) | | 1.29 (0.75, 2.23) | | | 0.352 | |  | | 0.82 (0.46, 1.46) | | 0.505 | | |  | 0.98 (0.54, 1.77) | | 0.947 | |
| Q4 [1368, 1787] | 36 (4.43%) | | 1.35 (0.79, 2.31) | | | 0.276 | |  | | 0.54 (0.29, 1.02) | | 0.057 | | |  | 0.79 (0.41, 1.52) | | 0.489 | |
| P for trend |  | | 0.080 | | | | |  | | 0.092 | | | | |  | 0.738 | | | |
| Age [65, 74] |  | |  | | |  | |  | |  | |  | | |  |  | |  | |
| BMR (kcal/day) |  | |  | | |  | |  | |  | |  | | |  |  | |  | |
| Per SD increase | 224 (8.64%) | | 0.99 (0.87, 1.14) | | | 0.940 | |  | | 0.77 (0.66, 0.91) | | 0.002 | | |  | 0.80 (0.67, 0.94) | | 0.007 | |
| Quartiles of BMR |  | |  | | |  | |  | |  | |  | | |  |  | |  | |
| Q1 [787, 1115] | 55 (7.49%) | | *Ref* | | |  | |  | | *Ref* | |  | | |  | *Ref* | |  | |
| Q2 [1116, 1219] | 57 (9.15%) | | 1.23 (0.84, 1.78) | | | 0.285 | |  | | 1.01 (0.69, 1.47) | | 0.970 | | |  | 1.05 (0.72, 1.54) | | 0.780 | |
| Q3 [1220, 1367] | 66 (9.97%) | | 1.37 (0.95, 1.95) | | | 0.088 | |  | | 0.93 (0.63, 1.37) | | 0.715 | | |  | 0.96 (0.65, 1.42) | | 0.850 | |
| Q4 [1368, 1781] | 46 (8.01%) | | 1.07 (0.72, 1.58) | | | 0.748 | |  | | 0.58 (0.37, 0.90) | | 0.015 | | |  | 0.63 (0.40, 1.00) | | 0.048 | |
| P for trend |  | | 0.708 | | | | |  | | 0.008 | | | | |  | 0.030 | | | |
| Age [75, 97] |  | |  | | |  | |  | |  | |  | | |  |  | |  | |
| BMR (kcal /day) |  | |  | | |  | |  | |  | |  | | |  |  | |  | |
| Per SD increase | 396 (22.50%) | | 0.99 (0.90, 1.10) | | | 0.916 | |  | | 0.79 (0.70, 0.89) | | <0.001 | | |  | 0.81 (0.71, 0.92) | | 0.001 | |
| Quartiles of BMR |  | |  | | |  | |  | |  | |  | | |  |  | |  | |
| Q1 [790, 1115] | 158 (22.16%) | | *Ref* | | |  | |  | | *Ref* | |  | | |  | *Ref* | |  | |
| Q2 [1116, 1219] | 98 (22.95%) | | 1.05 (0.82, 1.35) | | | 0.700 | |  | | 0.80 (0.61, 1.04) | | 0.092 | | |  | 0.81 (0.62, 1.05) | | 0.115 | |
| Q3 [1220, 1367] | 91 (23.88%) | | 1.07 (0.82, 1.38) | | | 0.628 | |  | | 0.68 (0.51, 0.90) | | 0.007 | | |  | 0.69 (0.51, 0.93) | | 0.013 | |
| Q4 [1368, 1788] | 49 (20.50%) | | 0.91 (0.66, 1.26) | | | 0.583 | |  | | 0.52 (0.36, 0.73) | | <0.001 | | |  | 0.56 (0.39, 0.80) | | 0.002 | |
| P for trend |  | | 0.749 | | | | |  | | <0.001 | | | | |  | <0.001 | | | |
| Abbreviations: BMR, basal metabolic rate; *Ref*, reference; *HR*, hazard ratio; *CI*, confidence interval; SD, standard deviation. Model Ⅰ adjusted for gender.  ModelⅡadjusted for sex, SBP, DBP, education level, current smokers and drinkers, physical activity levels, sleep duration on workdays or non-workdays, ACEI or ARB and calcium channel blockers usage. | | | | | | | | | | | | | | | | | | | |
